# Calcium signals in guard cells enhance the efficiency by which abscisic acid triggers stomatal closure

**DOI:** 10.1111/nph.15985

**Published:** 2019-07-19

**Authors:** Shouguang Huang, Rainer Waadt, Maris Nuhkat, Hannes Kollist, Rainer Hedrich, M. Rob G. Roelfsema

**Affiliations:** ^1^ Molecular Plant Physiology and Biophysics Julius‐von‐Sachs Institute for Biosciences Biocenter, Würzburg University Julius‐von‐Sachs‐Platz 2 D‐97082 Würzburg Germany; ^2^ Centre for Organismal Studies Plant Developmental Biology Ruprecht‐Karls‐Universität Heidelberg Im Neuenheimer Feld 230 D‐69120 Heidelberg Germany; ^3^ Institute of Technology University of Tartu Nooruse 1 Tartu 50411 Estonia

**Keywords:** abscisic acid (ABA), Ca^2+^‐indicator, cytosolic Ca^2+^ signals, OST1 protein kinase, R‐GECO1‐mTurquoise, SLAC1 and SLAH3 anion channels, stomata

## Abstract

- During drought, abscisic acid (ABA) induces closure of stomata via a signaling pathway that involves the calcium (Ca^2+^)‐independent protein kinase OST1, as well as Ca^2+^‐dependent protein kinases. However, the interconnection between OST1 and Ca^2+^ signaling in ABA‐induced stomatal closure has not been fully resolved.
- ABA‐induced Ca^2+^ signals were monitored in intact Arabidopsis leaves, which express the ratiometric Ca^2+^ reporter R‐GECO1‐mTurquoise and the Ca^2+^‐dependent activation of S‐type anion channels was recorded with intracellular double‐barreled microelectrodes.
- ABA triggered Ca^2+^ signals that occurred during the initiation period, as well as in the acceleration phase of stomatal closure. However, a subset of stomata closed in the absence of Ca^2+^ signals. On average, stomata closed faster if Ca^2+^ signals were elicited during the ABA response. Loss of OST1 prevented ABA‐induced stomatal closure and repressed Ca^2+^ signals, whereas elevation of the cytosolic Ca^2+^ concentration caused a rapid activation of SLAC1 and SLAH3 anion channels.
- Our data show that the majority of Ca^2+^ signals are evoked during the acceleration phase of stomatal closure, which is initiated by OST1. These Ca^2+^ signals are likely to activate Ca^2+^‐dependent protein kinases, which enhance the activity of S‐type anion channels and boost stomatal closure.

## Introduction

Land plants control gas exchange with the surrounding atmosphere by modulating the aperture of stomatal pores in the leaf surface (Shimazaki *et al*., 2007; Kim *et al*., 2010; Kollist *et al*., 2014). In the light, stomata open and enable CO_2_ uptake for photosynthesis, whereas they close during drought to protect plants from desiccation. Several lines of evidence show that the stress hormone abscisic acid (ABA) plays a central role in drought‐induced stomatal closure (Roelfsema *et al*., 2012; Munemasa *et al*., 2015). An in‐depth understanding of the molecular mechanisms that underlie ABA‐dependent stomatal closure, therefore, can open new strategies of breeding plants with improved drought tolerance.

Stomata rapidly close after stimulation with extracellular ABA (Guzel Deger *et al*., 2015), which was taken as an indication that this stress hormone is perceived by a cell surface receptor (Joshi‐Saha *et al*., 2011). However, recent findings point to a rapid uptake of ABA into guard cells (Boursiac *et al*., 2013; Merilo *et al*., 2015), which is followed by the perception through cytosolic PYrabactin Resistant/PYrabactin resistant‐Like/Regulatory Component of ABA Receptors (PYR/PYL/RCAR; Levchenko *et al*., 2008; Ma *et al*., 2009; Park *et al*., 2009). Loss of multiple of these PYR/PYL/RCAR receptors causes stomata to become ABA insensitive (Gonzalez‐Guzman *et al*., 2012; Merilo *et al*., 2013), which indicates that these proteins are essential for guard cell ABA perception.

A short signaling pathway leads to activation of the SLow Anion Channel 1 (SLAC1) in guard cells (Roelfsema *et al*., 2012; Munemasa *et al*., 2015; Hedrich & Geiger, 2017), in which the protein kinase Open STomata 1 (OST1) is a central player (Mustilli *et al*., 2002). In the absence of ABA, a group of class 2C protein phosphatases (PP2Cs; including ABA Insensitive 1 and 2) inhibits OST1 (Umezawa *et al*., 2009; Vlad *et al*., 2009). Binding of ABA to its PYR1/PYL/RCAR receptors causes them to deactivate the PP2Cs and thus release OST1 from inhibition (Ma *et al*., 2009; Park *et al*., 2009). Once OST1 gets activated, it phosphorylates and stimulates SLAC1, which leads to the extrusion of anions and causes a depolarization of the guard cell plasma membrane (Pei *et al*., 1997; Roelfsema *et al*., 2004; Geiger *et al*., 2009; Lee *et al*., 2009). As a result, depolarization‐dependent potassium (K^+^) channels are activated, anions and K^+^ are released by guard cells; this reduces their osmotic content and causes stomatal closure in less than 20 min (Kollist *et al*., 2014; Guzel Deger *et al*., 2015; Hedrich & Geiger, 2017).

In addition to the calcium (Ca^2+^)‐independent OST1 pathway, guard cells are also likely to exhibit a Ca^2+^‐dependent signaling chain that activates SLAC1, as well as the homologous channel SLAH3 (Geiger *et al*., 2011; Brandt *et al*., 2015; Guzel Deger *et al*., 2015). Such a Ca^2+^‐dependent pathway was already postulated in the pioneering work of De Silva *et al*. (1985). Later experiments with Ca^2+^‐sensitive dyes revealed that ABA can indeed trigger a transient elevation of the cytosolic free Ca^2+^ concentration in *Commelina communis* guard cells (McAinsh *et al*., 1990; Gilroy *et al*., 1991). The ABA‐dependent rise of the cytosolic Ca^2+^ level was postulated to activate plasma membrane anion channels, based on experiments in *Vicia faba*,* Arabidopsis* and tobacco (*Nicotiana tabacum*) guard cells (Schroeder & Hagiwara, 1989; Allen *et al*., 1999; Chen *et al*., 2010; Stange *et al*., 2010). However, the hypothesis was challenged by the contrasting finding that ABA is able to activate S‐type anion channels in the absence of cytosolic Ca^2+^ signals (Levchenko *et al*., 2005; Marten *et al*., 2007). One may thus propose that the guard cell ABA signaling pathway is based on a core Ca^2+^‐insensitive (OST1‐dependent) chain (Cutler *et al*., 2010), which is modulated by Ca^2+^‐dependent processes. However, the interconnection between these two branches and their individual roles in stomatal closure have not been resolved.

Most of the aforementioned guard cell studies that address Ca^2+^ signaling have been carried out with stomata in epidermal peels or epidermal fragments. These isolated tissues offer the advantage that fluorescence signals of guard cells are not disturbed by autofluorescence of mesophyll cells. Moreover, stimuli such as ABA can be easily applied to guard cells in epidermal peels, from the side that faces the leaf interior, whereas the side covered by the cuticle offers a strong barrier for many solutes. However, stomatal movements in epidermal peels are reduced in amplitude and response time, in comparison with the stomatal responses in intact leaves (Willmer & Mansfield, 1969; Roelfsema & Hedrich, 2002). It is thus desirable to work with guard cells in intact leaves, but this approach requires a new generation of reporters that enable cytosolic Ca^2+^ measurements in intact tissues.

Newly developed genetically encoded Ca^2+^‐ reporters, such as GCaMP6 and R‐GECO1, display much higher Ca^2+^‐dependent changes in fluorescence intensity, compared with Yellow Cameleon 3.6 (Zhao *et al*., 2011; Chen *et al*., 2013; Waadt *et al*., 2017). Recently, R‐GECO1 has been fused to mTurquoise to generate a highly sensitive Ca^2+^ sensor with an internal reference (Waadt *et al*., 2017). We therefore used intact Arabidopsis leaves that express R‐GECO1‐mTurquoise (RG‐mT) to study Ca^2+^ signals in stomata that were stimulated with ABA via microcapillaries in contact with the guard cell wall.

## Materials and Methods

### Plant material and growth conditions

All *Arabidopsis thaliana* lines were in the Col‐0 background, the *ost1‐3*,* slac1‐3, slah3‐1* single mutants, the *slac1‐3/slah3‐1* double mutants, and plants expressing RG‐mT have been described previously (Yoshida *et al*., 2002; Merilo *et al*., 2013; Guzel Deger *et al*., 2015; Waadt *et al*., 2017). The *ost1‐3* mutant was transformed with the RG‐mT construct, as described for wild‐type by Waadt *et al*. (2017), using the floral dip method and the *Agrobacterium tumefaciens* strain GV3101 (Zhang *et al*., 2006). Seeds were sown on sterilized soil, and plants were grown in a growth cabinet with 60% relative humidity, a cycle of 12 h : 12 h, light : dark, temperatures of 21°C (light) and 18°C (dark), and a photon flux density of 100 μmol m^−2^ s^−1^. After 12 d, the seedlings were transferred to pots (diameter 6 cm) and grown for another 2–3 wk in the same conditions.

Measurements were carried out on stomata, either in isolated epidermal peels or in intact leaves that were excised with a sharp razor blade from 4‐ to 5‐wk‐old plants and gently fixed in a petri dish (diameter 35 mm) with the adaxial side attached to double‐sided adhesive tape. The leaves were immersed in the following solution: 10 mM potassium chloride (KCl), 1 mM calcium chloride (CaCl_2_) and 10 mM potassium citrate, pH 5 and illuminated with white light (100 μmol m^−2^ s^−1^) for at least 2 h before the start of the experiment.

Epidermal strips were gently peeled with a pair of tweezers from the abaxial side of RG‐mT‐expressing leaves. The strips were fixed on cover slips (diameter 18 mm) with medical adhesive (Medical Adhesive B; Aromando, Düsseldorf, Germany) and placed in the following bath solution: 10 mM KCl, 1 mM CaCl_2_, 10 mM Mes–bis‐tris propane, pH 6.0.

### Microelectrode techniques

Stomata in the abaxial epidermis of intact leaves, or epidermal strips, were visualized with a water immersion objective (W Plan‐Apochromat, 63×/1.0; Carl Zeiss, Jena, Germany) mounted to an upright microscope (Axioskop 2FS; Zeiss). ABA was applied with single‐barreled microelectrodes that were pulled from borosilicate glass capillaries (inner diameter, 0.58 mm; outer diameter, 1.0 mm; Hilgenberg, Malsfeld, Germany; http://www.hilgenberg-gmbh.com) on a horizontal laser puller (P2000; Sutter Instruments Co., Novato, CA, USA). The electrode tips were filled with ABA at the standard concentration of 50 μM, or concentrations ranging from 0.5 to 100 μM to obtain a dose–response curve, and the electrodes then further filled with 300 mM KCl. Control experiments were conducted with 50 μM benzoic acid. The electrodes were connected via silver/silver chloride (Ag/AgCl) half‐cells to a headstage of a custom‐made amplifier (input impedance > 10^11^ Ω, Ulliclamp01). A glass capillary that was filled with 300 mM KCl and sealed with 300 mM KCl in 2% agarose served as a reference electrode. The microelectrodes were mounted to a piezo‐driven micro‐manipulator (MM3A; Kleindiek Nanotechnik, Reutlingen, Germany) and slowly moved towards the guard cell wall. The tip potential was monitored during manipulation of the microelectrode, and when the electrode came into contact with the guard cell wall it suddenly changed to values more negative than −15 mV. After establishment of a connection between the guard cell wall and microelectrode, ABA, or benzoic acid as control, was ejected from the electrode with a current of −0.8 nA for a period of 20–30 s. Directly after termination of current ejection, the microelectrode was removed from the guard cell wall.

The plasma membrane conductance of guard cells was studied in voltage clamp experiments with double‐barreled microelectrodes. These microelectrodes were fabricated from two borosilicate capillaries (inner diameter, 0.58 mm; outer diameter, 1.0 mm; Hilgenberg), which were aligned, heated, twisted 360°, and pre‐pulled on a vertical puller (L/M‐3P‐A; Heka, Lambrecht/Pfalz, Germany). Subsequently, the joint capillaries were pulled on a horizontal laser puller (P2000; Sutter Instruments Co.). The double‐barreled electrodes were backfilled with 300 mM KCl and had a tip resistance that ranged from 180 to 280 MΩ. Both barrels of the microelectrode were connected by Ag/AgCl half cells to the Ulliclamp01 amplifier, which enables voltage clamp experiments with an internal differential amplifier. Voltage pulses were applied with winwcp software (Dempster, 1997; University of Strathclyde, https://www.strath.ac.uk) and recorded at 1 kHz, using USB‐6002 interfaces (National Instruments, Austin, TX, USA; http://www.ni.com). A dual low‐pass Bessel filter (LPF 202A; Warner Instruments Corp., Hamden, CT, USA) was used to low‐pass filter the electrical signals at 0.5 kHz.

### Quantitative fluorescence microscopy

Fluorescence signals of Ca^2+^‐imaging experiments were obtained from regions of interest in the central part of guard cells that included the nucleus. The measurements were carried out with a charge‐multiplying charge‐coupled device camera (QuantEM; Photometrics; http://www.photometrics.com) that was mounted to a CARV, Crestoptics, Rome, Italy confocal spinning disc unit. Within the CARV unit, three filter wheels were used, while the spinning disc was moved out of the light path. The R‐GECO1 and mTurquoise subunits in RG‐mT were excited with light of an LED illumination system (pE‐4000; CoolLED, Andover, UK) at 435 nm and 580 nm, respectively. The emission signals were passed through dichroic mirrors with cut‐off wavelengths of 450 nm (T450 LPXR; Chroma Technology Corp., Bellows Falls, VT, USA) and 590 nm (FF593 BrightLine; Semrock, http://www.semrock.com) and band filters at 475/28 nm (BrightLine HC; Semrock, Semrock Inc., IDEX Corp.; Lake Forest, IL, USA) and 628/40 nm (BrightLine; Semrock).

Changes in stomatal aperture were monitored during the Ca^2+^‐imaging experiments, with light provided by a halogen bulb in the microscope lamp, filtered through a far‐red light bandpass filter (713/30 nm). All images were analyzed offline with the image‐j/fiji software package (Schindelin *et al*., 2012). The statistical and mathematical analysis of the data was carried out with prism 6 and 7 (GraphPad Software, San Diego, CA, USA; https://www.graphpad.com) and origin pro 8 (Originlab Corp., Northampton, MA, USA).

## Results

### ABA ejection evokes movement of single guard cells

**Figure 1:**
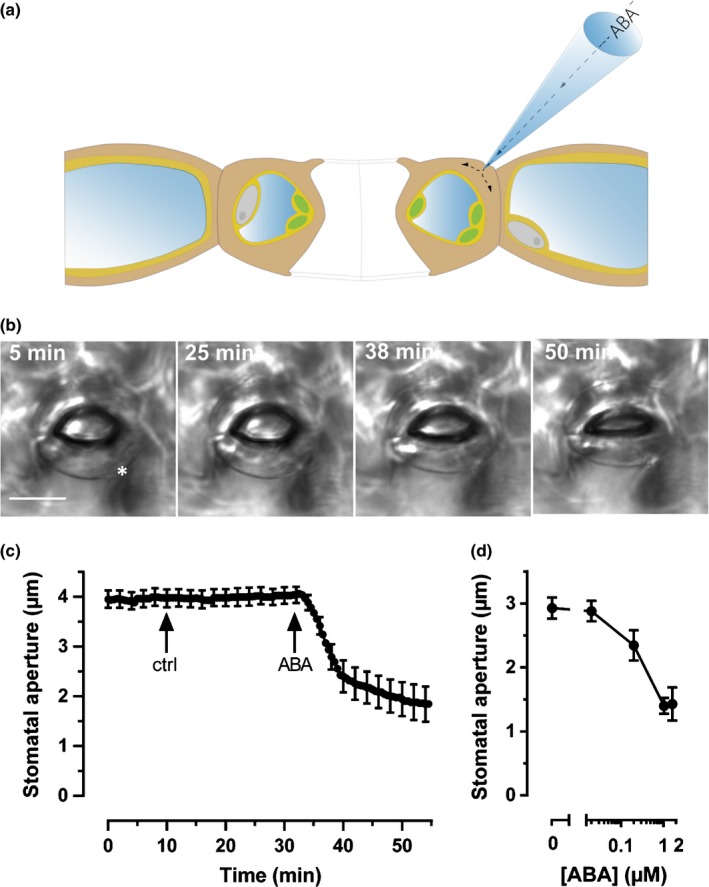
Current ejection of abscisic acid (ABA) induces rapid stomatal closure. (a) Cartoon of the current‐ejection technique. The tip of an electrode was filled with 50 μM ABA (or 50 μM benzoic acid as control) and brought in contact with the wall of a guard cell, in an intact *Arabidopsis thaliana* leaf. ABA was ejected via the microelectrode, by application of a −0.8 nA current. Stomatal movements were monitored with a charge‐coupled device camera mounted to an upright microscope. (b) Images of a stoma in an intact leaf, acquired before and after current ejection of benzoic acid, as a control (25 min), and ABA (38 and 50 min). The time points marked in the images correspond to the time axis shown in (c). The asterisk marks the position at which the microelectrode was in contact with the guard cell wall. Bar, 10 μm. Note that stomatal movement was mainly due to a change in shape of the guard cell close to the microelectrode, whereas the other guard cell remained bent. See also Supporting Information Videos S1. (c) Time‐dependent changes of the average stomatal aperture, after subsequent application of benzoic acid and ABA, as indicated by arrows. Note that current ejection of benzoic acid did not affect the stomatal aperture, whereas ABA triggered rapid stomatal closure. Average data are shown, ± SE, *n* = 9. (d) Dose–response curve of ABA‐induced stomatal closure. The dose of ABA was varied by modulating the hormone concentration in the current‐ejection electrode and estimated as explained in Methods S1.

In a previous study, rapid stomatal closure was induced by nanoinfusion of ABA‐containing solution, which was pressure‐injected through open stomata into intact leaves (Guzel Deger *et al*., 2015). Based on this approach, we found that 20 μM ABA induces closure of stomata within 20 min. However, nanoinfusion alters the optical properties of the leaf surface, which is a disadvantage when it is used in combination with quantitative fluorescence microscopy. We therefore introduced a ‘current‐ejection technique’ to stimulate single guard cells with ABA in intact Arabidopsis leaves (Fig. 1a). Single‐barreled electrodes were slowly moved towards the abaxial epidermis of an intact leaf until the electrode tip came into contact with the guard cell wall (Fig. 1a). In this configuration, electrically charged molecules, such as ABA^−^, can be ejected from the glass microcapillary into the guard cell wall by application of current pulses.

Stimulation with ABA, by a current of −0.8 nA for 20–30 s, caused a rapid reduction of the stomatal aperture (Supporting Information Videos S1). After a lag time of only 1.44 min (Fig. 1b,c; SE = 0.29 min, *n* = 9), the stomata closed with a maximal velocity of 0.28 μm min^−1^ (SE = 0.05 μm min^−1^, *n* = 9). By contrast, application of benzoic acid as control (−0.8 nA, 20–30 s) did not affect the aperture of stomata (Fig. 1b,c). ABA had a strong impact on the guard cell that was closely located to the tip of the current‐ejection electrode (asterisk in Fig. 1b, left panel), whereas the guard cell on the other side of the pore remained curved (Fig. 1b).

The asymmetric response of guard cells in a stomatal complex suggests that the current‐ejection method only provides ABA to a restricted area of the guard cell wall. This was studied by current ejection of the fluorescent dye Lucifer Yellow CH (LY) using the same conditions as already described for ABA (Fig. S1b,c; Methods S1; Videos S2). Indeed, current ejection of LY resulted in a localized fluorescence signal that decreased exponentially from the tip of the electrode (Fig. S1b,c). Based on the LY experiments, it was estimated that the current‐ejection procedure transferred a short dose of ABA with a local concentration of 1.1 μM (Fig. S1d). The dose of ABA that was applied to guard cells could be modulated by changing the ABA concentration in the current‐ejection electrodes. This revealed that a local ABA concentration of 0.2 μM triggered stomatal closure, with only half of the average magnitude, compared with 1.1 or 1.6 μM ABA (Fig. 1d), whereas guard cells did not respond to 0.02 μM ABA (Fig. 1d).

### ABA‐induced Ca^2+^ signals are associated with initiation and acceleration of stomatal closure

**Figure 2:**
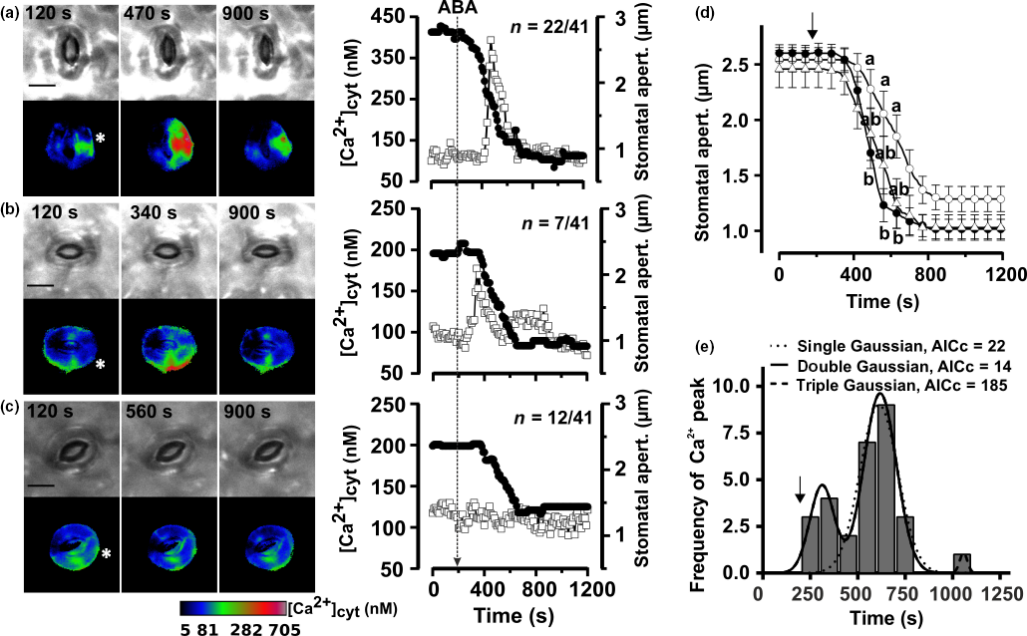
Abscisic acid (ABA)‐induced Ca^2+^ signals occur during the initiation or acceleration phase of stomatal closure and speed up stomatal closure. (a–c) Left panels: bright‐field images (upper images) showing the aperture of an *Arabidopsis thaliana* stoma and pseudo‐color images (lower images) showing the cytosolic Ca^2+^ concentration in guard cells of the same stoma. The images were obtained at three time points, as indicated in the bright‐field images. The asterisks in the pseudo‐color images indicate the position of contact between the current‐ejection electrode and the guard cell wall. The calibration bar below the images links the color code to [Ca^2+^]_cyt_. Bars, 10 μm. See also Supporting Information Videos S4–S6. Right panels: time‐dependent changes in stomatal aperture (closed circles) and the cytosolic free Ca^2+^ concentration (open squares) in guard cells that were stimulated by current ejection of ABA, as indicated by the arrow. Data are shown for representative cells, which either show (a) an increase of the cytosolic free Ca^2+^ concentration during stomatal closure (22 out of 41 cells), (b) before stomatal closure (7 out of 41 cells), or (c) no change in the cytosolic Ca^2+^ level (12 out of 41 cells). (d) ABA‐induced changes in stomatal aperture, averaged for stomata in which a Ca^2+^ signal occurred before stomatal closure (open triangles, *n* = 7), during stomatal closure (closed circles, *n* = 22), or without Ca^2+^ signals (open circles, *n* = 12). Arrow indicates the time point of ABA application; error bars represent ± SE. Data points at which the average stomatal aperture significantly differed between the groups of stomata are indicated by lower‐case letters. (e) Frequency distribution of the time period between stimulation with ABA and occurrence of a peak in the cytosolic Ca^2+^ level. The time period between current ejection of ABA and the maximal cytosolic Ca^2+^ concentration was calculated for the same cells as displayed in (a–c) and binned in intervals of 100 s. The frequency distribution was fitted with the sum of single (dotted line), double (solid line), and triple (striped line) Gaussian functions. Based on Akaike's information criterion (AICc), a model with two Gaussian functions (solid line) was 55 times more likely as one with a single Gaussian function (dotted line) and 10^37^ times more likely as one with three Gaussian functions (striped line). The arrow indicates the time point of current ejection of ABA.

Several genetically encoded fluorescent Ca^2+^ reporters have become available in recent years, of which RG‐mT was chosen, since it exhibits a strong Ca^2+^‐dependent change in fluorescence emission ratio and it does not dramatically affect plant growth (Zhao *et al*., 2011; Waadt *et al*., 2017). The emission ratio of RG‐mT was calibrated to the cytosolic Ca^2+^ concentration, using the fluorescent Ca^2+^‐reporter dye FURA2 (see Fig. S2; Methods S1; Videos S3), and far‐red light was used to monitor closure of the stomatal pore (Fig. 2a–c; Videos S4–S6). Current ejection of ABA caused closure of the stomatal pore in all experiments (*n* = 41); however, the impact of ABA on the cytosolic Ca^2+^ level split into three guard cell populations (Fig. 2a–c). In a first group of 22 out of 41 measured cells, ABA triggered a transient rise in the cytosolic free Ca^2+^ level during the phase in which closure of the stomata accelerated (Fig. 2a; Videos S4). In a second population of guard cells, the Ca^2+^ signal preceded stomatal closure (seven out of 41 cells; Fig. 2b; Videos S5). Finally, Ca^2+^ signals were lacking in guard cells during ABA‐induced stomatal closure in the remaining third population of 12 out of 41 cells (Fig. 2c; Videos S6). The occurrence of Ca^2+^ signals correlated with the speed of stomatal closure (Fig. 2d). Stomata in which Ca^2+^ signals occurred during stomatal closure reached the half‐maximal response in a significantly shorter period than stomata in which Ca^2+^ signals were absent (one‐way ANOVA, *P* = 0.002). On average, stomata with a transient Ca^2+^ rise displayed a half‐maximal closure within 289 s (SE = 19 s, *n* = 22), whereas this value was reached later (410 s, SE = 27 s, *n* = 12) in the absence of Ca^2+^ signals.

By contrast to the experiments with ABA, current ejection of benzoic acid did not cause stomatal closure in any of the 24 experiments. Only in two out of 24 guard cells were transient changes in the cytosolic free Ca^2+^ concentration observed, and these Ca^2+^ signals had a smaller amplitude than those elicited by ABA (Fig. S3).

Our data thus indicate that ABA‐induced Ca^2+^ signals can be clustered into two groups (Fig. 2a,b). In a small group, the Ca^2+^ signals precede stomatal closure (Fig. 2b), whereas the Ca^2+^ level rises during stomatal closure in the majority of guard cells (Fig. 2a). The occurrence of these groups was tested by fitting the frequency distribution of cells with Ca^2+^ signals; the number of cells were plotted against the time interval between stimulation with ABA and occurrence of the peak in the cytosolic Ca^2+^‐level (Fig. 2e). According to the corrected Akaike information criterion (Burnham *et al*., 2011), a model based on the sum of two Gaussian functions (solid line in Fig. 2e, *R* ^2^ = 0.99) was 55 times more likely than the model with one Gaussian function (dotted line in Fig. 2e, *R* ^2^ = 0.75), whereas a model based on the sums of three Gaussian functions was very unlikely (10^37^ less likely as the sum of two Gaussian functions; striped line in Fig. 2e, *R* ^2^ = 0.99). This analysis thus strongly supports that ABA‐induced Ca^2+^ signals in guard cells occur in two time windows; some are elicited early (before the stomata start to close), whereas others are evoked later (during stomatal closure).

### Elevated cytosolic Ca^2+^ levels rapidly activate S‐type anion channels

**Figure 3:**
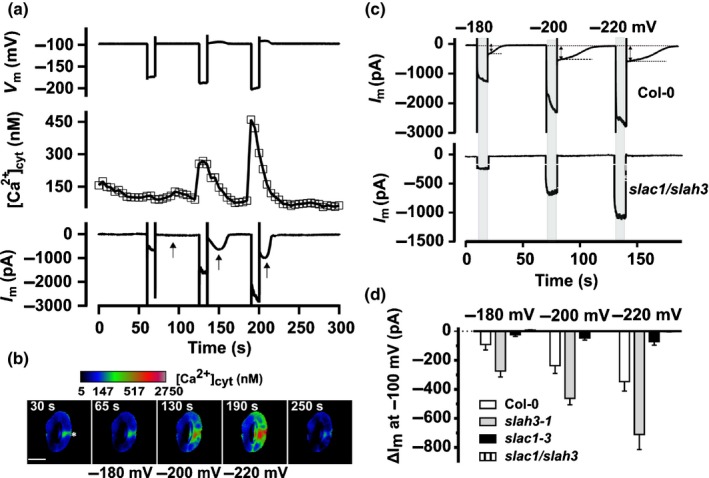
Cytosolic Ca^2+^ signals rapidly activate SLAC1 and SLAH3 anion channels in *Arabidopsis thaliana* guard cells. (a) Arabidopsis guard cells were stimulated with 10 s pulses from a potential of −100 mV, to −180, −200, and −220 mV (upper trace). The voltage pulses to −200 and −220 mV evoked a transient increase of the cytosolic free Ca^2+^ concentration (middle trace). The Ca^2+^ signals caused activation of inward currents (lower trace) after returning the voltage to −100 mV, as indicated by the arrows below the current trace. (b) Pseudo‐color images that represent the cytosolic free Ca^2+^ concentration of the same guard cell as in (a) determined from the R‐GECO1‐mTurquoise signal. The images were acquired before, during, and after the stimulation of the cell with hyperpolarizing voltage pulses. The asterisk marks the position at which the right guard cell was impaled with a double‐barreled electrode. Bar, 10 μm. The calibration bar above the images links the color code to [Ca^2+^]_cyt_. See also Supporting Information Videos S7. (c) Guard cells were stimulated with voltage pulses from a holding potential of −100 mV, for 10 s to −180, −200, and −220 mV as indicated above the current traces. In wild‐type guard cells these voltage pulses caused activation of anion channels (upper trace) that facilitate inward currents after returning the voltage to −100 mV, as indicated by the dotted lines. These anion currents were absent in guard cells of the *slac1‐3/slah3‐1* double mutant (lower trace). (d) Average currents facilitated by S‐type anion channels, as measured in (c), evoked by voltage pulses of −180, −200, and −220 mV in Col‐0 wild‐type (white bars), *slah3‐1* (gray bars; see also Fig. S4), *slac1‐3* (black bars; see also Fig. S4), and *slac1/slah3* (striped bars, note that bars approximate 0 pA). Errors bars represent + SE (Col‐0, *n* = 9; *slah3‐1*,* n* = 8; *slac1‐3*,* n* = 10; *slac1/slah3*,* n* = 13).

Elevated cytosolic Ca^2+^ levels were shown to activate plasma membrane anion channels in guard cells of several species (Allen *et al*., 1999; Chen *et al*., 2010; Stange *et al*., 2010), but it is unknown how fast this response occurs in Arabidopsis. We therefore studied this response in real time, with guard cells expressing RG‐mT. Guard cells were impaled with double‐barreled electrodes, and cytosolic Ca^2+^ concentration changes were evoked with voltage pulses (Grabov & Blatt, 1998; Voss *et al*., 2018) from −100 mV, stepwise for 10 s, to more negative membrane potentials (Fig. 3a,b). The cytosolic Ca^2+^ level of the cell shown in Fig. 3(a,b) hardly changed in response to a 10 s pulse of −180 mV, but a transient rise in the Ca^2+^ concentration was triggered by pulses to −200 and −220 mV (Videos S7). During the hyperpolarizing pulses, inward currents are facilitated by K^+^ channels, which are voltage activated and deactivate at −100 mV in *c*. 0.5 s (Roelfsema & Prins, 1997). After termination of the voltage pulses in which Ca^2+^ signals were elicited, an additional conductance was recorded (arrows in Fig. 3a), which transiently reached maximum conductance, at 13.8 s (SE = 0.9 s, *n* = 27) after the cytosolic Ca^2+^ peak (Fig. 3a). It is likely that this slow current is facilitated by Ca^2+^‐activated S‐type anion channels, as was previously shown for tobacco guard cells (Chen *et al*., 2010; Stange *et al*., 2010).

In Arabidopsis, guard cell S‐type anion channels are encoded by SLAC1 and SLAH3 (Negi *et al*., 2008; Vahisalu *et al*., 2008; Guzel Deger *et al*., 2015), and the voltage responses of the *slac1* and *slah3* loss‐of‐function mutants were therefore compared with wild‐type (Fig. 3c,d). In wild‐type, 10 s pulses from −100 mV, stepwise to −180, −200, and −220 mV, induced inward currents that slowly deactivated after returning to −100 mV (Fig. 3c), just as in guard cells expressing RG‐mT (Fig. 3a). In the *slah3‐1* single mutant, the hyperpolarizing pulses elicited currents that had a similar magnitude as in wild‐type (Figs 3d, S4). However, these currents were only detected in six out of 10 *slac1‐3* guard cells, where, on average, they had a reduced magnitude (Figs 3d, S4). Finally, the loss of both SLAC1 and SLAH3 caused a complete lack of Ca^2+^‐activated currents (Fig. 3c,d). These data thus strongly suggest that both SLAC1 and SLAH3 contribute to the Ca^2+^‐activated conductance in Arabidopsis guard cells.

### Loss of OST1 prevents ABA‐induced stomatal closure and alters Ca^2+^ signals

**Figure 4:**
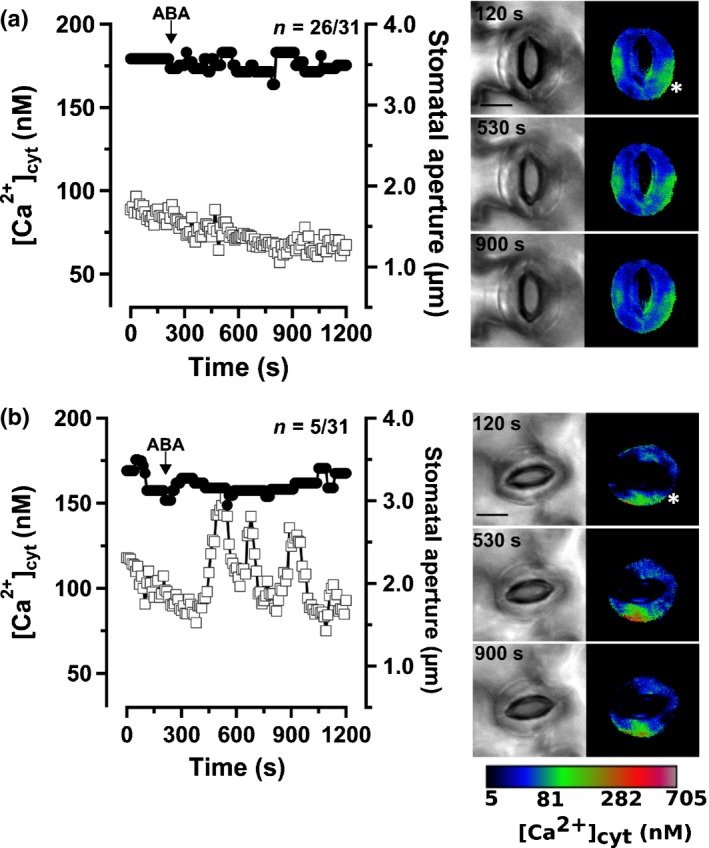
Abscisic acid (ABA) evokes Ca^2+^ signals only in five out of 31 *Arabidopsis thaliana ost1‐3* guard cells. (a, b) Left panels: stomatal aperture (closed circles) and the cytosolic free Ca^2+^ concentration (open squares) plotted against time of guard cells stimulated by current ejection of ABA, as indicated by the arrow. None of the stomata closed in response to ABA. (a) In 26 out of 31 cells, the guard cells did not display a change in the cytosolic free Ca^2+^ concentration. (b) ABA triggered repetitive rises in the cytosolic free Ca^2+^ level in five out of 31 cells; nevertheless, the stomata remained open. (a, b) Right panels: bright‐field (left image) and pseudo‐color (right image) images, showing the stomatal aperture and cytosolic free Ca^2+^ concentration in the same stomata as in the graphs on the left. The images were obtained at three time points, as indicated in the bright‐field images. The asterisks in the pseudo‐color images indicate the position of contact between the current‐ejection electrode and the guard cell wall. The calibration bar below the images links the color code to [Ca^2+^]_cyt_. Bars, 10 μm. See also Supporting Information Videos S8 & S9.

The protein kinase OST1 plays a central role in ABA‐induced stomatal closure (Mustilli *et al*., 2002; Merilo *et al*., 2013; Guzel Deger *et al*., 2015). However, it is unclear how loss of OST1 affects Ca^2+^ signals. Guard cells of *ost1‐3*, expressing RG‐mT, were therefore stimulated by current‐ejection of ABA. In the majority of guard cells, ABA neither induced stomatal closure nor provoked a change in cytosolic free Ca^2+^ level (Fig. 4a, 26 out of 31 cells; Videos S8). Despite the lack of stomatal closure, transient changes of the cytosolic free Ca^2+^ concentration were observed in five out of 31 guard cells (Fig. 4b; Videos S9). ABA thus triggered Ca^2+^ signals in approximately one out of six *ost1‐3* guard cells, whereas it evoked Ca^2+^ signals in three out of four guard cells of wild‐type (Fig. 2). For comparison, current ejection of benzoic acid as control evoked only a Ca^2+^ signal in one out of 21 *ost1‐3* guard cells (Fig. S5).

### Cytosolic Ca^2+^ signals trigger rapid activation of anion channels in *ost1‐3*

**Figure 5:**
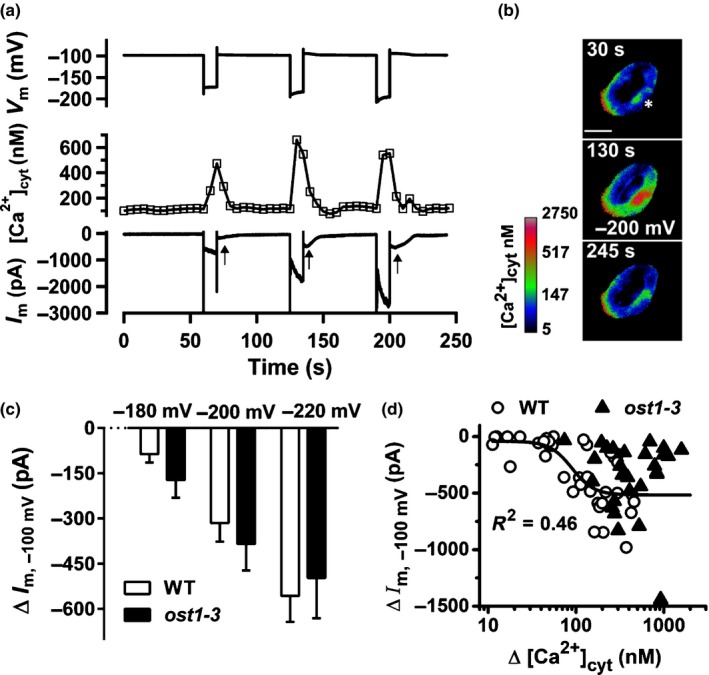
Ca^2+^‐dependent activation of S‐type anion channels in *ost1‐3*. (a) An *Arabidopsis thaliana ost1‐3* guard cell was stimulated with 10 s voltage pulses from a potential of −100 mV, to −180, −200 and −220 mV (upper trace). The voltage pulses evoked a transient increase of the cytosolic free Ca^2+^ concentration (middle traces), which caused activation of S‐type anion channels (lower trace) that facilitate inward currents after returning the voltage to −100 mV (arrows below the current trace). (b) Pseudo‐color images that represent the cytosolic free Ca^2+^ concentration of the same guard cell as in (a), determined from the R‐GECO1‐mTurquoise signal. The images were acquired before, during, and after application of the −200 mV voltage pulse. The asterisk marks the position at which the right guard cell was impaled with a double‐barreled electrode. Bar, 10 μm. The calibration bar next to the images links the color code to [Ca^2+^]_cyt_. See also Supporting Information Videos S10. (c) Average change in S‐type anion channel current, recorded at −100 mV and induced by hyperpolarizing pulses to −180, −200, and −220 mV, in wild type (WT, white bars) and *ost1‐*3 (black bars). Data are from experiments shown in Fig. 3(a) (WT, *n* = 13) and Fig. 5(a) (*ost1‐3*,* n* = 9). Error bars represent + SE. (d) Currents carried by S‐type anion channels, plotted against the peak in the cytosolic Ca^2+^ concentration, induced by voltage pulses. Data were obtained from 37 voltage pulses applied to 13 WT (open circles) guard cells and 27 voltage pulses in nine guard cells of *ost1‐3* (closed triangles). The WT data were fitted with a Hill function, which revealed a half‐maximal response at a change of the cytosolic Ca^2+^ concentration of 90 nM (SE = 26 nM) and a maximal anion channel current of −516 pA (SE = 74 pA). The Hill function did not converge with the data of *ost1‐3*.

OST1 is important for a variety of stomatal responses (Melotto *et al*., 2006; Xue *et al*., 2011; Merilo *et al*., 2013), but it is unclear to what extent it is necessary for Ca^2+^‐dependent responses in guard cells. Guard cells of *ost1‐3*, expressing RG‐mT, were therefore stimulated with 10 s hyperpolarization pulses (Fig. 5a,b). Just as in wild‐type, these pulses evoked a transient elevation of the cytosolic Ca^2+^ level (Videos S10) and activated S‐type anion channels, with a similar voltage dependence as in wild‐type (Fig. 5c). The cytosolic Ca^2+^ concentration changes were plotted against the currents carried by S‐type anion channels at −100 mV in Fig. 5(d). For wild‐type, a Hill equation was fitted to the data, which revealed that a 90 nM increase of the cytosolic Ca^2+^ concentration led to a half‐maximal response (Fig. 5d). The Hill equation did not converge to the data of *ost1‐3*, but the number of cells in which large changes of the cytosolic Ca^2+^ level occurred was higher in the mutant (Fig. 5d). Combined with the finding that the voltage pulses triggered S‐type anion channel currents with a similar magnitude in *ost1‐3* and wild‐type (Fig. 5c), this suggests that *ost1‐3* guard cells have a slightly lower Ca^2+^ responsiveness, as wild‐type.

## Discussion

ABA evoked stomatal closure in Arabidopsis in the absence of Ca^2+^ signals in one out of four stomata, whereas a transient rise in the Ca^2+^ level was detected in three out of four experiments. These data are in line with early experiments with *C. communis*, in which ABA‐dependent Ca^2+^ signals were detected in eight out of 10 stomata (McAinsh *et al*., 1990) or in 14 out of 38 stomata (Gilroy *et al*., 1991). This suggests that ABA‐induced Ca^2+^ signals are common in guard cells, but not absolutely required for stomatal closure.

### Ca^2+^ signals occur in two phases of the guard cell ABA response

The cytosolic Ca^2+^ signals arose in two phases after stimulation of Arabidopsis guard cells with ABA (Fig. 2). In the majority of cells, the cytosolic Ca^2+^ concentration increased transiently during the stage in which the stomata were closing. It is feasible that these Ca^2+^ signals are provoked by the sudden changes in osmotic content of guard cells, which arise at the start of stomatal closure. Such a mechanism is supported by the finding that fast changes in the osmotic content of tobacco guard cells provoke Ca^2+^ release from intracellular stores (Voss *et al*., 2016). This class of ABA‐induced Ca^2+^ signals will not occur in the *ost1‐3* mutant, as its stomata do not close in response to ABA, and thus osmotic changes in the cytosol are not evoked by the hormone. As a result, ABA‐induced Ca^2+^ signals are impaired in *ost1‐3* and only five out of 31 *ost1‐3* stomata showed changes in the cytosolic Ca^2+^ level; all of which did not exceed 100 nM (Fig. 4).

ABA can also induce Ca^2+^ signals that precede closure of the stomatal pore (Fig. 2b), which suggests that the hormone also stimulates Ca^2+^ channels by a mechanism that does not depend on changes in osmotic pressure. This early response may explain why ABA can also trigger repetitive rises in the Ca^2+^ concentration of Arabidopsis guard cells in isolated epidermal tissue (Allen *et al*., 1999, 2001; Klüsener *et al*., 2002; Islam *et al*., 2010). Note that in isolated epidermal tissues the ABA‐induced stomatal closure response is less pronounced than in intact leaves (Islam *et al*., 2010), and osmotically induced Ca^2+^ signals are therefore less likely to occur. This suggests that ABA evokes these early Ca^2+^ signals through a mechanism that is not dependent on OST1, but instead through stimulation of nonselective cation channels in the guard cell plasma membrane (Hamilton *et al*., 2000; Pei *et al*., 2000; Siegel *et al*., 2009).

### Role of Ca^2+^ signals in ABA‐induced stomatal closure

ABA‐induced stomatal closure is likely to involve a Ca^2+^‐independent and ‐dependent signaling mechanisms. The initial Ca^2+^‐independent step releases the protein kinase OST1 from inhibition (Cutler *et al*., 2010). In guard cells, OST1 will activate SLAC1, which leads to the release of anions from guard cells and provokes stomatal closure (Geiger *et al*., 2009; Lee *et al*., 2009). The Ca^2+^ signals that can occur before or during stomatal closure probably enhance the activity of SLAC1 and also activate SLAH3, since these two anion channels are also activated by hyperpolarization‐induced Ca^2+^ signals (Fig. 3). Owing to a further stimulation of the S‐type anion channels in guard cells, Ca^2+^ signals seem to speed up stomatal closure (Fig. 2d).

The Ca^2+^‐dependent response is likely to be provoked by Ca^2+^‐dependent protein kinases (CPKs; Harper *et al*., 1991; Geiger *et al*., 2010, 2011; Brandt *et al*., 2015) and calcineurin B‐like (CBL)‐interacting protein kinases (CIPKs) that bind to CBL proteins (Maierhofer *et al*., 2014; Kudla *et al*., 2018). Studies with CPK loss‐of‐function mutants support the function of these protein kinases in ABA‐induced stomatal closure. In *cpk8*,* cpk10*, the *cpk3/6* double, and the *cpk5/6/11/23* quadruple mutants, ABA‐induced stomatal closure was impaired in intact leaves that were floated on solution (Mori *et al*., 2006; Brandt *et al*., 2015; Zou *et al*., 2015). However, experiments with the *cpk23* and *cpk4/5/6/11* mutants put this general role of CPKs in question. ABA could still induce stomatal closure in the *cpk4/5/6/11* mutant (Guzel Deger *et al*., 2015), and loss of CPK23 even caused plants to become more tolerant to drought (Ma & Wu, 2007). Future studies will thus have to disclose which targets are addressed by individual CPKs and CIPKs and how these interactions contribute to the regulation of stomatal movements.

Cytosolic Ca^2+^ signals regulate not only plasma membrane ion channels but also the vacuolar two‐pore K^+^ channels, which are important for stomatal closure (Gobert *et al*., 2007; Latz *et al*., 2013; Wang *et al*., 2015). As suggested by Wheeler & Brownlee (2008), the Ca^2+^ signals may thus serve as a unifying signal that can coordinate transport processes between the plasma membrane and intracellular membranes. Such a coordinated response is likely to be important for rapid stomatal closure, in which osmolytes are first released from the vacuole into the cytosol and finally extruded across the plasma membrane into the apoplast (Wheeler & Brownlee, 2008; Kollist *et al*., 2014).

### Future directions

In addition to the drought hormone ABA, stomata also respond to a variety of other signals, such as CO_2_, microbe‐associated molecular patterns, and blue light. Previously, the associated Ca^2+^ signals were studied in isolated epidermal tissues (Young *et al*., 2006; Harada & Shimazaki, 2009; Thor & Peiter, 2014), but new genetically encoded Ca^2+^ sensors now enable experiments with intact leaves. Studies with these new sensors can reveal if guard cell Ca^2+^ responses are stimulus specific, or if similar Ca^2+^ signals are recorded, irrespective of the stimulus that induces stomatal closure.

The newly developed sensors will also be of great advantage to study the nature of Ca^2+^ channels that give rise to Ca^2+^ signals in guard cells. ABA‐induced activation of Ca^2+^‐permeable plasma membrane channels in guard cells was reported almost 20 yr ago (Hamilton *et al*., 2000; Pei *et al*., 2000), but the genes encoding these channels still need to be uncovered. The osmotically acitavated calcium channels, which are expressed in guard cells, have been associated with osmotically induced Ca^2+^ signals (Yuan *et al*., 2014). These channels are thus good candidates for those that generate Ca^2+^ signals during acceleration phase of ABA‐induced stomatal closure.

## Author contributions

RW, RH, and MRGR initiated and designed the study, SH and MN performed the experiments, SH, MN, and MRGR conducted the data analysis and prepared the figures, and SH, HK, RW, RH, and MRGR wrote the manuscript.

## Supporting information

Please note: Wiley Blackwell are not responsible for the content or functionality of any Supporting Information supplied by the authors. Any queries (other than missing material) should be directed to the *New Phytologist* Central Office.

**Fig. S1** Quantification of ABA concentrations evoked by current‐ejection.
**Fig. S2** Calibration of RG‐mT with FURA2 in *Arabidopsis* guard cells.
**Fig. S3** Current‐ejection of benzoic acid only induces Ca^2+^ signals in 2 out of 24 guard cells.
**Fig. S4** Inward currents triggered by hyperpolarization of wild type, *slah3‐1* and *slac1‐3* guard cells.
**Fig. S5** Current‐ejection of benzoic acid only induces Ca^2+^ signals in 1 out of 21 *ost1‐3* guard cells.

**Methods S1** Procedures to estimate the ABA concentration in the guard cell wall that was imposed by current‐ejection and calibration of R‐GECO1‐mTurquoise with FURA2.

**Videos S1** Stomatal closure induced by current‐ejection of ABA.
**Videos S2** Current‐ejection of Lucifer Yellow CH (LY) into the wall of an *Arabidopsis* guard cell.
**Videos S3** Calibration of R‐GECO1‐mTuquiose (RG‐mT) with FURA2.
**Videos S4** ABA‐induced rise in the cytosolic Ca^2+^ concentration of a guard cell, during stomatal closure.
**Videos S5** ABA‐induced rise in the cytosolic Ca^2+^ concentration of a guard cell, before stomatal closure.
**Videos S6** ABA‐induced stomatal closure in the absence of a cytosolic Ca^2+^ signal in the guard cells.
**Videos S7** Voltage‐induced Ca^2+^ signals in an *Arabidopsis* guard cell.
**Videos S8** *ost1‐3* stoma exposed to ABA, which did neither evoke stomatal closure, nor Ca^2+^‐signals.
**Videos S9** ABA‐induced Ca^2+^‐signals in an *ost1‐3* stoma that were not linked to stomatal closure.
**Videos S10** Voltage‐induced Ca^2+^ signals in an *ost1‐3* guard cell.

## References

[nph15985-bib-0001] Allen GJ , Chu SP , Harrington CL , Schumacher K , Hoffman T , Tang YY , Grill E , Schroeder JI . 2001 A defined range of guard cell calcium oscillation parameters encodes stomatal movements. Nature 411: 1053–1057. 11429606 10.1038/35082575

[nph15985-bib-0002] Allen GJ , Kuchitsu K , Chu SP , Murata Y , Schroeder JI . 1999 Arabidopsis *abi1‐1* and *abi2‐1* phosphatase mutations reduce abscisic acid‐induced cytoplasmic calcium rises in guard cells. Plant Cell 11: 1785–1798. 10488243 10.1105/tpc.11.9.1785 PMC144302

[nph15985-bib-0003] Boursiac Y , Leran S , Corratge‐Faillie C , Gojon A , Krouk G , Lacombe B . 2013 ABA transport and transporters. Trends in Plant Science 18: 325–333. 23453706 10.1016/j.tplants.2013.01.007

[nph15985-bib-0004] Brandt B , Munemasa S , Wang C , Nguyen D , Yong TM , Yang PG , Poretsky E , Belknap TF , Waadt R , Aleman F *et al* 2015 Calcium specificity signaling mechanisms in abscisic acid signal transduction in *Arabidopsis* guard cells. eLife 4: e03599. 10.7554/eLife.03599 PMC4507714 26192964

[nph15985-bib-0005] Burnham KP , Anderson DR , Huyvaert KP . 2011 AIC model selection and multimodel inference in behavioral ecology: some background, observations, and comparisons. Behavioral Ecology and Sociobiology 65: 23–35.

[nph15985-bib-0006] Chen ZH , Hills A , Lim CK , Blatt MR . 2010 Dynamic regulation of guard cell anion channels by cytosolic free Ca^2+^ concentration and protein phosphorylation. The Plant Journal 61: 816–825. 20015065 10.1111/j.1365-313X.2009.04108.x

[nph15985-bib-0007] Chen TW , Wardill TJ , Sun Y , Pulver SR , Renninger SL , Baohan A , Schreiter ER , Kerr RA , Orger MB , Jayaraman V *et al* 2013 Ultrasensitive fluorescent proteins for imaging neuronal activity. Nature 499: 295–300. 23868258 10.1038/nature12354 PMC3777791

[nph15985-bib-0008] Cutler SR , Rodriguez PL , Finkelstein RR , Abrams SR . 2010 Abscisic acid: emergence of a core signaling network. Annual Review of Plant Biology 61: 651–679. 10.1146/annurev-arplant-042809-112122 20192755

[nph15985-bib-0009] De Silva DLR , Hetherington AM , Mansfield TA . 1985 Synergism between calcium ion and abscisic acid in preventing stomatal opening. New Phytologist 100: 473–482.

[nph15985-bib-0010] Dempster J . 1997 A new version of the Strathclyde electrophysiology software package running within the Microsoft windows environment. Journal of Physiology 504P: P57.

[nph15985-bib-0011] Geiger D , Maierhofer T , Al‐Rasheid KAS , Scherzer S , Mumm P , Liese A , Ache P , Wellmann C , Marten I , Grill E *et al* 2011 Stomatal closure by fast abscisic acid signaling is mediated by the guard cell anion channel SLAH3 and the receptor RCAR1. Science Signaling 4: ra32. 21586729 10.1126/scisignal.2001346

[nph15985-bib-0012] Geiger D , Scherzer S , Mumm P , Marten I , Ache P , Matschi S , Liese A , Wellmann C , Al‐Rasheid KAS , Grill E *et al* 2010 Guard cell anion channel SLAC1 is regulated by CDPK protein kinases with distinct Ca^2+^ affinities. Proceedings of the National Academy of Sciences, USA 107: 8023–8028. 10.1073/pnas.0912030107 PMC2867891 20385816

[nph15985-bib-0013] Geiger D , Scherzer S , Mumm P , Stange A , Marten I , Bauer H , Ache P , Matschi S , Liese A , Al‐Rasheid KAS *et al* 2009 Activity of guard cell anion channel SLAC1 is controlled by drought‐stress signaling kinase–phosphatase pair. Proceedings of the National Academy of Sciences, USA 106: 21425–21430. 10.1073/pnas.0912021106 PMC2795561 19955405

[nph15985-bib-0014] Gilroy S , Fricker MD , Read ND , Trewayas AJ . 1991 Role of calcium in signal transduction of *Commelina* guard cells. Plant Cell 3: 333–344. 12324599 10.1105/tpc.3.4.333 PMC160004

[nph15985-bib-0015] Gobert A , Isayenkov S , Voelker C , Czempinski K , Maathuis FJM . 2007 The two‐pore channel *TPK1* gene encodes the vacuolar K^+^ conductance and plays a role in K^+^ homeostasis. Proceedings of the National Academy of Sciences, USA 104: 10726–10731. 10.1073/pnas.0702595104 PMC1965580 17563365

[nph15985-bib-0016] Gonzalez‐Guzman M , Pizzio GA , Antoni R , Vera‐Sirera F , Merilo E , Bassel GW , Fernandez MA , Holdsworth MJ , Perez‐Amador MA , Kollist H *et al* 2012 *Arabidopsis* PYR/PYL/RCAR receptors play a major role in quantitative regulation of stomatal aperture and transcriptional response to abscisic acid. Plant Cell 24: 2483–2496. 22739828 10.1105/tpc.112.098574 PMC3406898

[nph15985-bib-0017] Grabov A , Blatt MR . 1998 Membrane voltage initiates Ca^2+^ waves and potentiates Ca^2+^ increases with abscisic acid in stomatal guard cells. Proceedings of the National Academy of Sciences, USA 95: 4778–4783. 10.1073/pnas.95.8.4778 PMC22567 9539815

[nph15985-bib-0018] Guzel Deger A , Scherzer S , Nuhkat M , Kedzierska J , Kollist H , Brosche M , Unyayar S , Boudsocq M , Hedrich R , Roelfsema MRG . 2015 Guard cell SLAC1‐type anion channels mediate flagellin‐induced stomatal closure. New Phytologist 208: 162–173. 25932909 10.1111/nph.13435 PMC4949714

[nph15985-bib-0019] Hamilton DWA , Hills A , Kohler B , Blatt MR . 2000 Ca^2+^ channels at the plasma membrane of stomatal guard cells are activated by hyperpolarization and abscisic acid. Proceedings of the National Academy of Sciences, USA 97: 4967–4972. 10.1073/pnas.080068897 PMC18341 10781106

[nph15985-bib-0020] Harada A , Shimazaki K . 2009 Measurement of changes in cytosolic Ca^2+^ in *Arabidopsis* guard cells and mesophyll cells in response to blue light. Plant and Cell Physiology 50: 360–373. 19106118 10.1093/pcp/pcn203

[nph15985-bib-0021] Harper JF , Sussman MR , Schaller GE , Putnamevans C , Charbonneau H , Harmon AC . 1991 A calcium‐dependent protein‐kinase with a regulatory domain similar to calmodulin. Science 252: 951–954. 1852075 10.1126/science.1852075

[nph15985-bib-0022] Hedrich R , Geiger D . 2017 Biology of SLAC1‐type anion channels – from nutrient uptake to stomatal closure. New Phytologist 216: 46–61. 28722226 10.1111/nph.14685

[nph15985-bib-0023] Islam MM , Munemasa S , Hossain MA , Nakamura Y , Mori IC , Murata Y . 2010 Roles of *At*TPC1, vacuolar Two Pore Channel 1, in *Arabidopsis* stomatal closure. Plant and Cell Physiology 51: 302–311. 20061305 10.1093/pcp/pcq001

[nph15985-bib-0024] Joshi‐Saha A , Valon C , Leung J . 2011 A brand new START: abscisic acid perception and transduction in the guard cell. Science Signaling 4: re4. 22126965 10.1126/scisignal.2002164

[nph15985-bib-0025] Kim TH , Böhmer M , Hu HH , Nishimura N , Schroeder JI . 2010 Guard cell signal transduction network: advances in understanding abscisic acid, CO_2_, and Ca^2+^ signaling. Annual Review of Plant Biology 61: 561–591. 10.1146/annurev-arplant-042809-112226 PMC3056615 20192751

[nph15985-bib-0026] Klüsener B , Young JJ , Murata Y , Allen GJ , Mori IC , Hugouvieux V , Schroeder JI . 2002 Convergence of calcium signaling pathways of pathogenic elicitors and abscisic acid in *Arabidopsis* guard cells. Plant Physiology 130: 2152–2163. 12481099 10.1104/pp.012187 PMC166727

[nph15985-bib-0027] Kollist H , Nuhkat M , Roelfsema MRG . 2014 Closing gaps: linking elements that control stomatal movement. New Phytologist 203: 44–62. 24800691 10.1111/nph.12832

[nph15985-bib-0028] Kudla J , Becker D , Grill E , Hedrich R , Hippler M , Kummer U , Parniske M , Romeis T , Schumacher K . 2018 Advances and current challenges in calcium signaling. New Phytologist 218: 414–431. 29332310 10.1111/nph.14966

[nph15985-bib-0029] Latz A , Mehlmer N , Zapf S , Mueller TD , Wurzinger B , Pfister B , Csaszar E , Hedrich R , Teige M , Becker D . 2013 Salt stress triggers phosphorylation of the *Arabidopsis* vacuolar K channel TPK1 by calcium‐dependent protein kinases (CDPKs). Molecular Plant 6: 1274–1289. 23253603 10.1093/mp/sss158 PMC3971370

[nph15985-bib-0030] Lee SC , Lan WZ , Buchanan BB , Luan S . 2009 A protein kinase–phosphatase pair interacts with an ion channel to regulate ABA signaling in plant guard cells. Proceedings of the National Academy of Sciences, USA 106: 21419–21424. 10.1073/pnas.0910601106 PMC2795491 19955427

[nph15985-bib-0031] Levchenko V , Guinot DR , Klein M , Roelfsema MRG , Hedrich R , Dietrich P . 2008 Stringent control of cytoplasmic Ca^2+^ in guard cells of intact plants compared to their counterparts in epidermal strips or guard cell protoplasts. Protoplasma 233: 61–72. 18648729 10.1007/s00709-008-0307-x

[nph15985-bib-0032] Levchenko V , Konrad KR , Dietrich P , Roelfsema MRG , Hedrich R . 2005 Cytosolic abscisic acid activates guard cell anion channels without preceding Ca^2+^ signals. Proceedings of the National Academy of Sciences, USA 102: 4203–4208. 10.1073/pnas.0500146102 PMC554796 15753314

[nph15985-bib-0033] Ma Y , Szostkiewicz I , Korte A , Moes D , Yang Y , Christmann A , Grill E . 2009 Regulators of PP2C phosphatase activity function as abscisic acid sensors. Science 324: 1064–1068. 19407143 10.1126/science.1172408

[nph15985-bib-0034] Ma SY , Wu WH . 2007 AtCPK23 functions in *Arabidopsis* responses to drought and salt stresses. Plant Molecular Biology 65: 511–518. 17541706 10.1007/s11103-007-9187-2

[nph15985-bib-0035] Maierhofer T , Diekmann M , Offenborn JN , Lind C , Bauer H , Hashimoto K , Al‐Rasheid KAS , Luan S , Kudla J , Geiger D *et al* 2014 Site‐ and kinase‐specific phosphorylation‐mediated activation of SLAC1, a guard cell anion channel stimulated by abscisic acid. Science Signaling 7: ra86. 25205850 10.1126/scisignal.2005703

[nph15985-bib-0036] Marten H , Konrad KR , Dietrich P , Roelfsema MRG , Hedrich R . 2007 Ca^2+^‐dependent and ‐independent abscisic acid activation of plasma membrane anion channels in guard cells of *Nicotiana tabacum* . Plant Physiology 143: 28–37. 17142476 10.1104/pp.106.092643 PMC1761993

[nph15985-bib-0037] McAinsh MR , Brownlee C , Hetherington AM . 1990 Abscisic acid‐induced elevation of guard‐cell cytosolic Ca^2+^ precedes stomatal closure. Nature 343: 186–188.

[nph15985-bib-0038] Melotto M , Underwood W , Koczan J , Nomura K , He SY . 2006 Plant stomata function in innate immunity against bacterial invasion. Cell 126: 969–980. 16959575 10.1016/j.cell.2006.06.054

[nph15985-bib-0039] Merilo E , Jalakas P , Kollist H , Brosche M . 2015 The role of ABA recycling and transporter proteins in rapid stomatal responses to reduced air humidity, elevated CO_2_, and exogenous ABA. Molecular Plant 8: 657–659. 25620768 10.1016/j.molp.2015.01.014

[nph15985-bib-0040] Merilo E , Laanemets K , Hu H , Xue S , Jakobsen L , Tulva I , Gonzales‐Guzman M , Rodriguez PL , Schroeder JI , Brosche M *et al* 2013 PYR/RCAR receptors contribute to ozone‐, reduced air humidity‐, darkness‐ and CO_2_‐induced stomatal regulation. Plant Physiology 162: 1652–1668. 23703845 10.1104/pp.113.220608 PMC3707544

[nph15985-bib-0041] Mori IC , Murata Y , Yang YZ , Munemasa S , Wang YF , Andreoli S , Tiriac H , Alonso JM , Harper JF , Ecker JR *et al* 2006 CDPKs CPK6 and CPK3 function in ABA regulation of guard cell S‐type anion‐ and Ca^2+^‐permeable channels and stomatal closure. PLoS Biology 4: 1749–1762. 10.1371/journal.pbio.0040327 PMC1592316 17032064

[nph15985-bib-0042] Munemasa S , Hauser F , Park J , Waadt R , Brandt B , Schroeder JI . 2015 Mechanisms of abscisic acid‐mediated control of stomatal aperture. Current Opinion in Plant Biology 28: 154–162. 26599955 10.1016/j.pbi.2015.10.010 PMC4679528

[nph15985-bib-0043] Mustilli AC , Merlot S , Vavasseur A , Fenzi F , Giraudat J . 2002 Arabidopsis OST1 protein kinase mediates the regulation of stomatal aperture by abscisic acid and acts upstream of reactive oxygen species production. Plant Cell 14: 3089–3099. 12468729 10.1105/tpc.007906 PMC151204

[nph15985-bib-0044] Negi J , Matsuda O , Nagasawa T , Oba Y , Takahashi H , Kawai‐Yamada M , Uchimiya H , Hashimoto M , Iba K . 2008 CO_2_ regulator SLAC1 and its homologues are essential for anion homeostasis in plant cells. Nature 452: 483–486. 18305482 10.1038/nature06720

[nph15985-bib-0045] Park SY , Fung P , Nishimura N , Jensen DR , Fujii H , Zhao Y , Lumba S , Santiago J , Rodrigues A , Chow TFF *et al* 2009 Abscisic acid inhibits type 2C protein phosphatases via the PYR/PYL family of START proteins. Science 324: 1068–1071. 19407142 10.1126/science.1173041 PMC2827199

[nph15985-bib-0046] Pei ZM , Kuchitsu K , Ward JM , Schwarz M , Schroeder JI . 1997 Differential abscisic acid regulation of guard cell slow anion channels in *Arabidopsis* wild‐type and *abi1* and *abi2* mutants. Plant Cell 9: 409–423. 9090884 10.1105/tpc.9.3.409 PMC156927

[nph15985-bib-0047] Pei ZM , Murata Y , Benning G , Thomine S , Klusener B , Allen GJ , Grill E , Schroeder JI . 2000 Calcium channels activated by hydrogen peroxide mediate abscisic acid signalling in guard cells. Nature 406: 731–734. 10963598 10.1038/35021067

[nph15985-bib-0048] Roelfsema MRG , Hedrich R . 2002 Studying guard cells in the intact plant: modulation of stomatal movement by apoplastic factors. New Phytologist 153: 425–431. 10.1046/j.0028-646X.2001.Documedoc.doc.x 33863230

[nph15985-bib-0049] Roelfsema MRG , Hedrich R , Geiger D . 2012 Anion channels: master switches of stress responses. Trends in Plant Science 17: 221–229. 22381565 10.1016/j.tplants.2012.01.009

[nph15985-bib-0050] Roelfsema MRG , Levchenko V , Hedrich R . 2004 ABA depolarizes guard cells in intact plants, through a transient activation of R‐ and S‐type anion channels. The Plant Journal 37: 578–588. 14756768 10.1111/j.1365-313x.2003.01985.x

[nph15985-bib-0051] Roelfsema MRG , Prins HBA . 1997 Ion channels in guard cells of *Arabidopsis thaliana* (L.) Heynh. Planta 202: 18–27. 9177048 10.1007/s004250050098

[nph15985-bib-0052] Schindelin J , Arganda‐Carreras I , Frise E , Kaynig V , Longair M , Pietzsch T , Preibisch S , Rueden C , Saalfeld S , Schmid B *et al* 2012 fiji: an open‐source platform for biological‐image analysis. Nature Methods 9: 676–682. 22743772 10.1038/nmeth.2019 PMC3855844

[nph15985-bib-0053] Schroeder JI , Hagiwara S . 1989 Cytosolic calcium regulates ion channels in the plasma‐membrane of *Vicia faba* guard‐cells. Nature 338: 427–430.

[nph15985-bib-0054] Shimazaki KI , Doi M , Assmann SM , Kinoshita T . 2007 Light regulation of stomatal movement. Annual Review of Plant Biology 58: 219‐247. 10.1146/annurev.arplant.57.032905.105434 17209798

[nph15985-bib-0055] Siegel RS , Xue SW , Murata Y , Yang YZ , Nishimura N , Wang A , Schroeder JI . 2009 Calcium elevation‐dependent and attenuated resting calcium‐dependent abscisic acid induction of stomatal closure and abscisic acid‐induced enhancement of calcium sensitivities of S‐type anion and inward‐rectifying K^+^ channels in *Arabidopsis* guard cells. The Plant Journal 59: 207–220. 19302418 10.1111/j.1365-313X.2009.03872.x PMC2827207

[nph15985-bib-0056] Stange A , Hedrich R , Roelfsema MRG . 2010 Ca^2+^‐dependent activation of guard cell anion channels, triggered by hyperpolarization, is promoted by prolonged depolarization. The Plant Journal 62: 265–276. 20088896 10.1111/j.1365-313X.2010.04141.x

[nph15985-bib-0057] Thor K , Peiter E . 2014 Cytosolic calcium signals elicited by the pathogen‐associated molecular pattern flg22 in stomatal guard cells are of an oscillatory nature. New Phytologist 204: 873–881. 25243759 10.1111/nph.13064

[nph15985-bib-0058] Umezawa T , Sugiyama N , Mizoguchi M , Hayashi S , Myouga F , Yamaguchi‐Shinozaki K , Ishihama Y , Hirayama T , Shinozaki K . 2009 Type 2C protein phosphatases directly regulate abscisic acid‐activated protein kinases in *Arabidopsis* . Proceedings of the National Academy of Sciences, USA 106: 17588–17593. 10.1073/pnas.0907095106 PMC2754379 19805022

[nph15985-bib-0059] Vahisalu T , Kollist H , Wang YF , Nishimura N , Chan WY , Valerio G , Lamminmaki A , Brosche M , Moldau H , Desikan R *et al* 2008 SLAC1 is required for plant guard cell S‐type anion channel function in stomatal signalling. Nature 452: 487–491. 18305484 10.1038/nature06608 PMC2858982

[nph15985-bib-0060] Vlad F , Rubio S , Rodrigues A , Sirichandra C , Belin C , Robert N , Leung J , Rodriguez PL , Lauriere C , Merlot S . 2009 Protein phosphatases 2C regulate the activation of the Snf1‐related kinase OST1 by abscisic acid in *Arabidopsis* . Plant Cell 21: 3170–3184. 19855047 10.1105/tpc.109.069179 PMC2782292

[nph15985-bib-0061] Voss LJ , Hedrich R , Roelfsema MRG . 2016 Current injection provokes rapid expansion of the guard cell cytosolic volume and triggers Ca^2+^ signals. Molecular Plant 9: 471–480. 26902185 10.1016/j.molp.2016.02.004

[nph15985-bib-0062] Voss LJ , McAdam SAM , Knoblauch M , Rathje JM , Brodribb TJ , Hedrich R , Roelfsema MRG . 2018 Guard cells in fern stomata are connected by plasmodesmata, but control cytosolic Ca^2+^ levels autonomously. New Phytologist 219: 206–215. 29655174 10.1111/nph.15153

[nph15985-bib-0063] Waadt R , Krebs M , Kudla J , Schumacher K . 2017 Multiparameter imaging of calcium and abscisic acid and high‐resolution quantitative calcium measurements using R‐GECO1‐mTurquoise in *Arabidopsis* . New Phytologist 216: 303–320. 28850185 10.1111/nph.14706

[nph15985-bib-0064] Wang Y , Dindas J , Rienmuller F , Krebs M , Waadt R , Schumacher K , Wu W‐H , Hedrich R , Roelfsema MRG . 2015 Cytosolic Ca^2+^ signals enhance the vacuolar ion conductivity of bulging *Arabidopsis* root hair cells. Molecular Plant 8: 1665–1674. 26232520 10.1016/j.molp.2015.07.009

[nph15985-bib-0065] Wheeler GL , Brownlee C . 2008 Ca^2+^ signalling in plants and green algae – changing channels. Trends in Plant Science 13: 506–514. 18703378 10.1016/j.tplants.2008.06.004

[nph15985-bib-0066] Willmer CM , Mansfield TA . 1969 A critical examination of use of detached epidermis in studies of stomatal physiology. New Phytologist 68: 363–375.

[nph15985-bib-0067] Xue SW , Hu HH , Ries A , Merilo E , Kollist H , Schroeder JI . 2011 Central functions of bicarbonate in S‐type anion channel activation and OST1 protein kinase in CO_2_ signal transduction in guard cell. EMBO Journal 30: 1645–1658. 21423149 10.1038/emboj.2011.68 PMC3102275

[nph15985-bib-0068] Yoshida R , Hobo T , Ichimura K , Mizoguchi T , Takahashi F , Aronso J , Ecker JR , Shinozaki K . 2002 ABA‐activated SnRK2 protein kinase is required for dehydration stress signaling in *Arabidopsis* . Plant and Cell Physiology 43: 1473–1483. 12514244 10.1093/pcp/pcf188

[nph15985-bib-0069] Young JJ , Mehta S , Israelsson M , Godoski J , Grill E , Schroeder JI . 2006 CO_2_ signaling in guard cells: calcium sensitivity response modulation, a Ca^2+^‐independent phase, and CO_2_ insensitivity of the *gca2* mutant. Proceedings of the National Academy of Sciences, USA 103: 7506–7511. 10.1073/pnas.0602225103 PMC1464368 16651523

[nph15985-bib-0070] Yuan F , Yang HM , Xue Y , Kong DD , Ye R , Li CJ , Zhang JY , Theprungsirikul L , Shrift T , Krichilsky B *et al* 2014 OSCA1 mediates osmotic‐stress‐evoked Ca^2+^ increases vital for osmosensing in *Arabidopsis* . Nature 514: 367–371. 25162526 10.1038/nature13593

[nph15985-bib-0071] Zhang XR , Henriques R , Lin SS , Niu QW , Chua NH . 2006 *Agrobacterium*‐mediated transformation of *Arabidopsis thaliana* using the floral dip method. Nature Protocols 1: 641–646. 17406292 10.1038/nprot.2006.97

[nph15985-bib-0072] Zhao YX , Araki S , Jiahui WH , Teramoto T , Chang YF , Nakano M , Abdelfattah AS , Fujiwara M , Ishihara T , Nagai T *et al* 2011 An expanded palette of genetically encoded Ca^2+^ indicators. Science 333: 1888–1891. 21903779 10.1126/science.1208592 PMC3560286

[nph15985-bib-0073] Zou JJ , Li XD , Ratnasekera D , Wang C , Liu WX , Song LF , Zhang WZ , Wu WH . 2015 Arabidopsis CALCIUM‐DEPENDENT PROTEIN KINASE8 and CATALASE3 function in abscisic acid‐mediated signaling and H_2_O_2_ homeostasis in stomatal guard cells under drought stress. Plant Cell 27: 1445–1460. 25966761 10.1105/tpc.15.00144 PMC4456645

