# Supplementary material for: Calcium signals in guard cells enhance the efficiency by which abscisic acid triggers stomatal closure
Source: New Phytol. 2019 Jul 19;224(1):177–87. doi: 10.1111/nph.15985 (PMC6771588; doi:10.1111/nph.15985)
Supplement: Supplementary file 1 — Fig. S1 Quantification of ABA concentrations evoked by current‐ejection. Fig. S2 Calibration of RG‐mT with FURA2 in Arabidopsis guard cells. Fig. S3 Current‐ejection of benzoic acid only induces Ca2+ signals in 2 out of 24 guard cells. Fig. S4 Inward currents triggered by hyperpolarization of wild type, slah3‐1 and slac1‐3 guard cells. Fig. S5 Current‐ejection of benzoic acid only induces Ca2+ signals in 1 out of 21 ost1‐3 guard cells. [file NPH-224-177-s001.pdf]

Supporting Information Figures S1-S5

Ca^{2+} signals in guard cells enhance the efficiency by which ABA triggers stomatal closure

By: Shouguang Huang, Rainer Waadt, Maris Nuhkat, Hannes Kollist, Rainer Hedrich and M. Rob G. Roelfsema

Article acceptance date: 3 June 2019

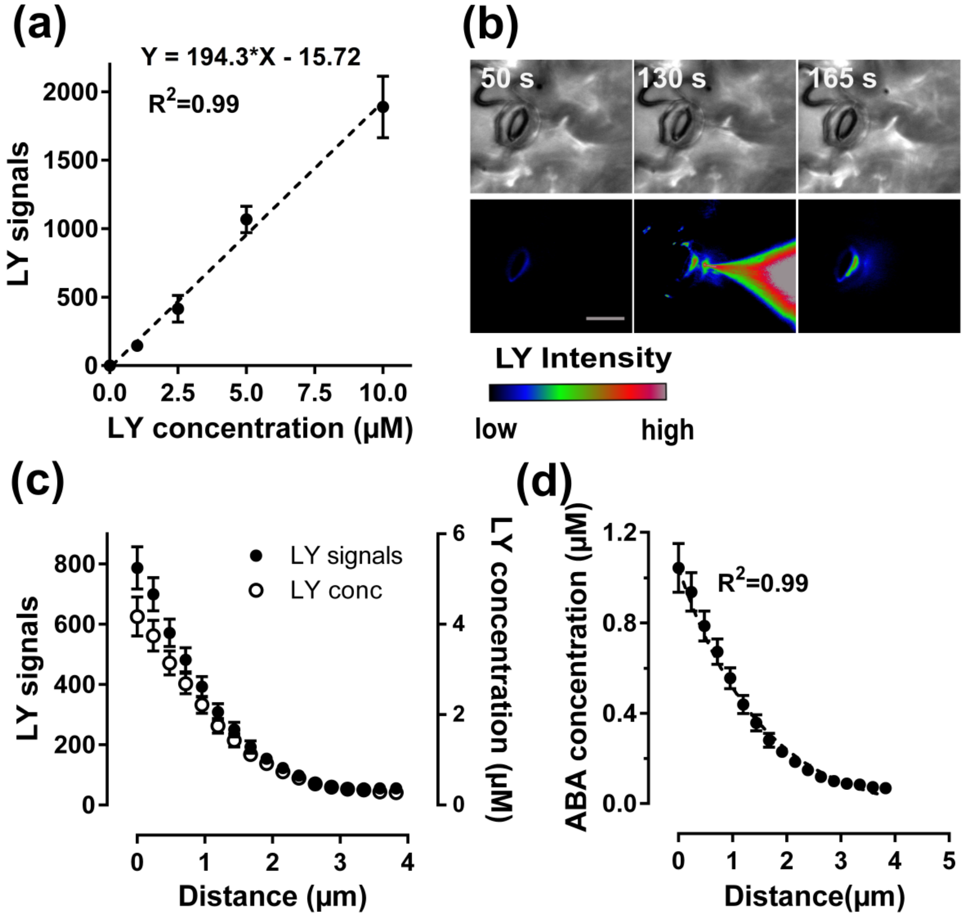

Fig. S1 Quantification of ABA concentrations evoked by current-ejection (see also Supporting Information Methods S1). (a) Calibration of the fluorescence microscope setup for the emission signal (520 nm) of Lucifer Yellow CH (LY). LY, at a range of concentrations, was pressure ejected from capillaries with an opening of 1 µM. A linear relation was obtained between the signal detected by the CCD camera at the tip of the capillaries and the LY concentration in the capillary. (b) Current-ejection of LY with a current of -0.8 nA over a period of 30s, from an electrode filled with 50 µM LY in the tip. *Upper panels*, transmitted light images of stoma in intact *Arabidopsis thaliana* leaf. The time (s) from the start of the experiment is indicated in each image. Lower panels, false colored images that indicate the LY fluorescence signal. Current ejection of LY was started at t=100 s and caused a local rise in the LY signal close to the tip of the electrode, which decreased in time after termination of current-ejection (see also Video S2). (c) Average LY signals imposed to the guard cell wall by current-ejection with -0.8 nA over a period of 30s. The LY concentration at the end of current application (t=130 s) is plotted against the distance from the tip of the electrode. (d) The ABA concentration gradient from the electrode tip, which is evoked by current-ejection into guard cell walls. The ABA concentrations were calculated with use of the LY signals (c), as explained in the Supporting Information Methods S1 and fitted with a single exponential equation.

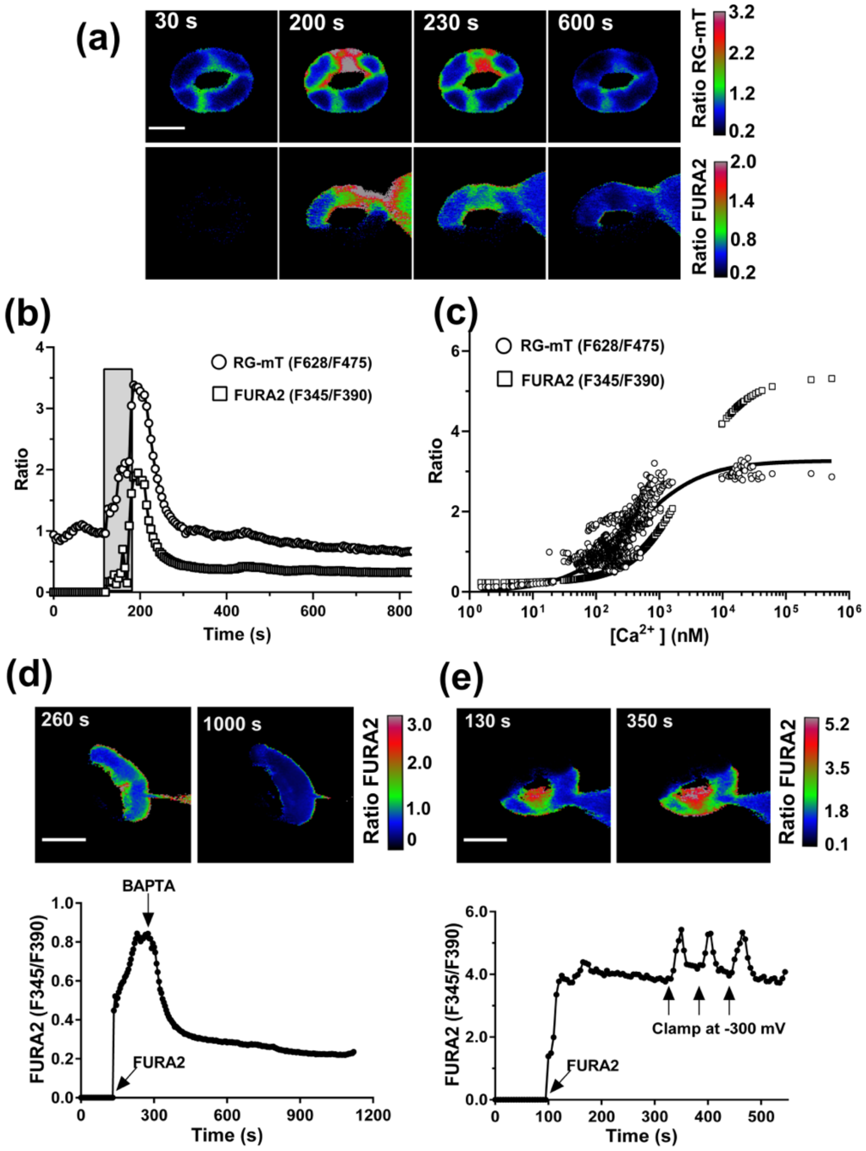

Fig. S2 Calibration of RG-mT with FURA2 in *Arabidopsis thaliana* guard cells (see also Supporting Information Methods S1). (a) Pseudo-color images representing the fluorescence emission ratio value (628/475 nm) of RG-mT (top panel) and excitation ratio (345/390 nm) of FURA2 (bottom panel) in an *Arabidopsis* guard cell. The cell was impaled with a double-barreled microelectrode and current-injected with FURA2 from 120 to 180 s, as shown in (b). The images were recorded before (30 s) and at three timepoints after (200, 230 and 600s) injection of FURA2 into the upper guard cell, using a current of -200 pA. The color-codes of RG-mT and FURA2 are linked to the respective ratios, by the calibration bars on the right. The scale bar represents 10 µm. See also Video S3. (b) Time-dependent changes of RG-mT (circles) and FURA2 (squares) ratio signals in the upper guard cell in (a). Note, that the ratio signals of RG-mT and FURA2 increased simultaneously during current injection of FURA2 (indicated by the grey area), whereas these ratio values slowly returned to pre-stimulus values after termination of current injection. (c) Fluorescence ratio values of RG-mT (circles) and FURA2 (squares) plotted against the cytosolic free Ca^{2+} concentration (logarithmic scale) in guard cells, which were calculated from the FURA2 signal. The FURA2 and RG-mT signals were measured simultaneously in *Arabidopsis* guard cells (n= 2179 from 21 guard cells), as shown in (a) and (b). A Hill equation was fitted to the RG-mT data, which revealed a Hill coefficient of 0.81 and a K_d of 401 nM (SE=17). (d and e) Calibration of the FURA2 signal with minimal and maximal values of the fluorescence excitation (345/390 nm) ratio. *Upper panels*, false colored images that indicate the FURA2 ratio in an *Arabidopsis* guard cells. *Lower panels*, time-dependent changes in the FURA2 ratio of the same cells as in the upper panels. The cells were first injected with FURA2 and thereafter injected with BAPTA (d) or stimulated with hyperpolarizing pulses of 10s to -300 mV (e). Arrows indicate the time points at which FURA2 or BAPTA were current injected, or at which the cell was clamped to -300 mV. Note, that the injection of BAPTA lowered the FURA2 ratio to a value of 0.23 (SE=0.002, n=6), whereas the hyperpolarizing pulses increased the FURA2 ratio to an average value of 5.34 (SE=0.16, n=6).

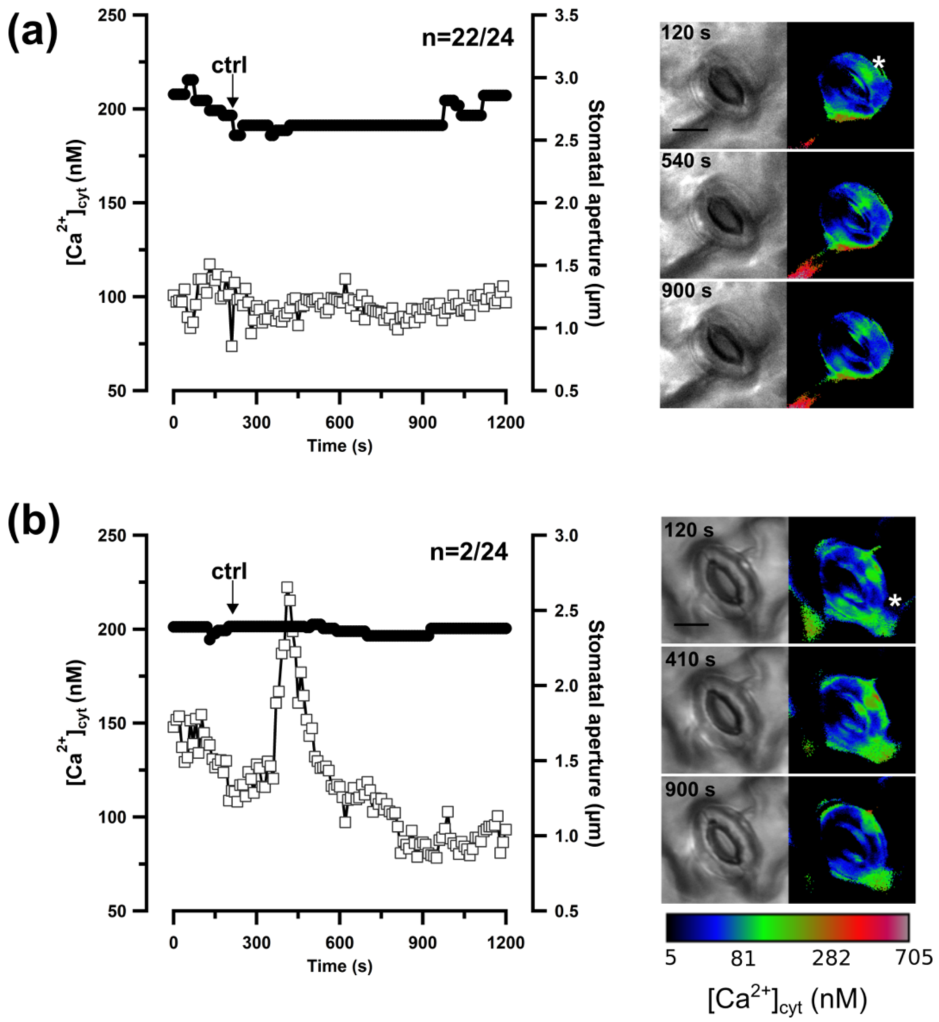

Fig. S3 Current-ejection of benzoic acid only induces Ca^{2+} signals in 2 out of 24 *Arabidopsis thaliana* guard cells. (a and b) *left panels*, Time-dependent changes in stomatal aperture (closed circles) and the cytosolic free Ca^{2+} concentration (open squares) in guard cells, stimulated by current-ejection of benzoic acid (control), as indicated by the arrow. Data are shown for representative guard cells. (a) In 22 out of 24 guard cells, current-ejection with benzoic acid neither caused stomatal closure, nor a change in the cytosolic free Ca^{2+} concentration. (b) Benzoic acid triggered an increase in the cytosolic free Ca^{2+} level in 2 out of 24 cells, but nevertheless the stomata remain open. (a, b) *right panels*, Bright field (left image) and pseudo-color (right image) images, showing the stomatal aperture and cytosolic free Ca^{2+} concentration of the same guard cell, as shown in graphs on the left. The images were obtained at three time points, as indicated in the bright field images. The asterisks in the pseudo-color images indicate the position of contact between the current-ejection electrode and the guard cell wall. The calibration bar below the images links the color-code to the cytosolic Ca^{2+} concentration. Scale bar=10 µm.

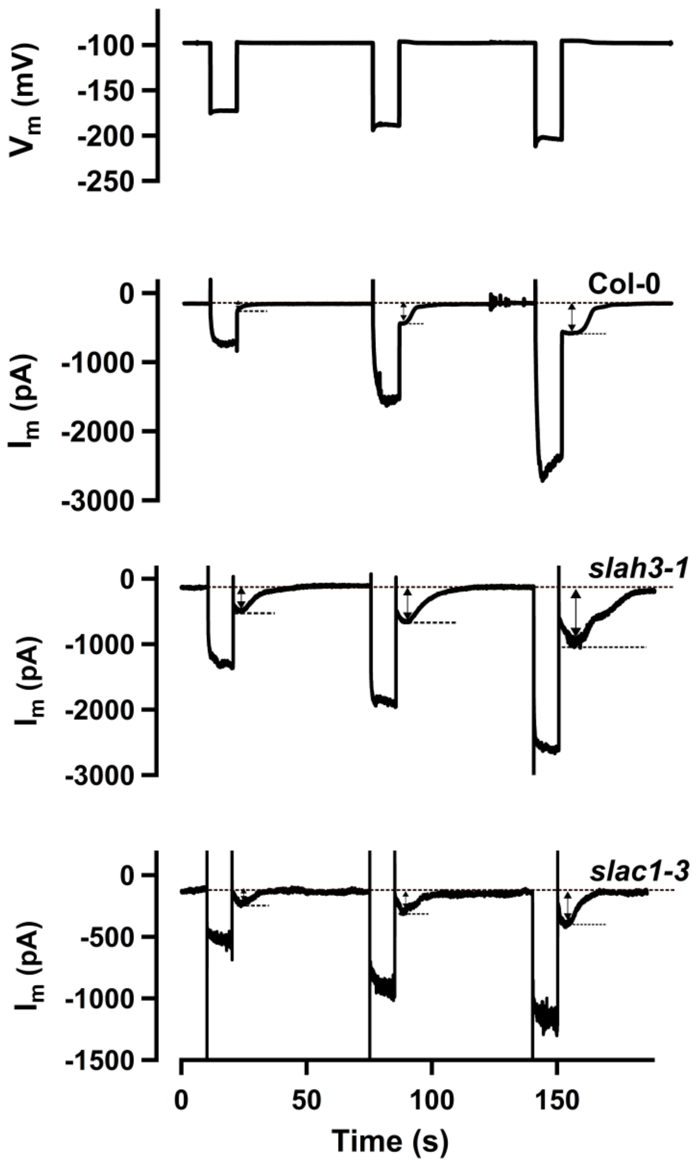

Fig. S4 Inward currents triggered by hyperpolarization of wild type *Arabidopsis thaliana*, *slah3-1* and *slac1-3* guard cells. Guard cells were stimulated with voltage pulses from a holding potential of -100 mV, for 10 s to -180, -200 and -220 mV (upper trace). In wild type guard cells these voltage pulses caused activation of anion channels (second trace from above) that facilitate inward currents after returning the voltage to -100 mV, as indicated by the dotted lines. Similar currents were observed in *slah3-1* guard cells (third trace from above), whereas this conductance was only observed in 6 out 10 *slac1-3* guard cells (lower trace).

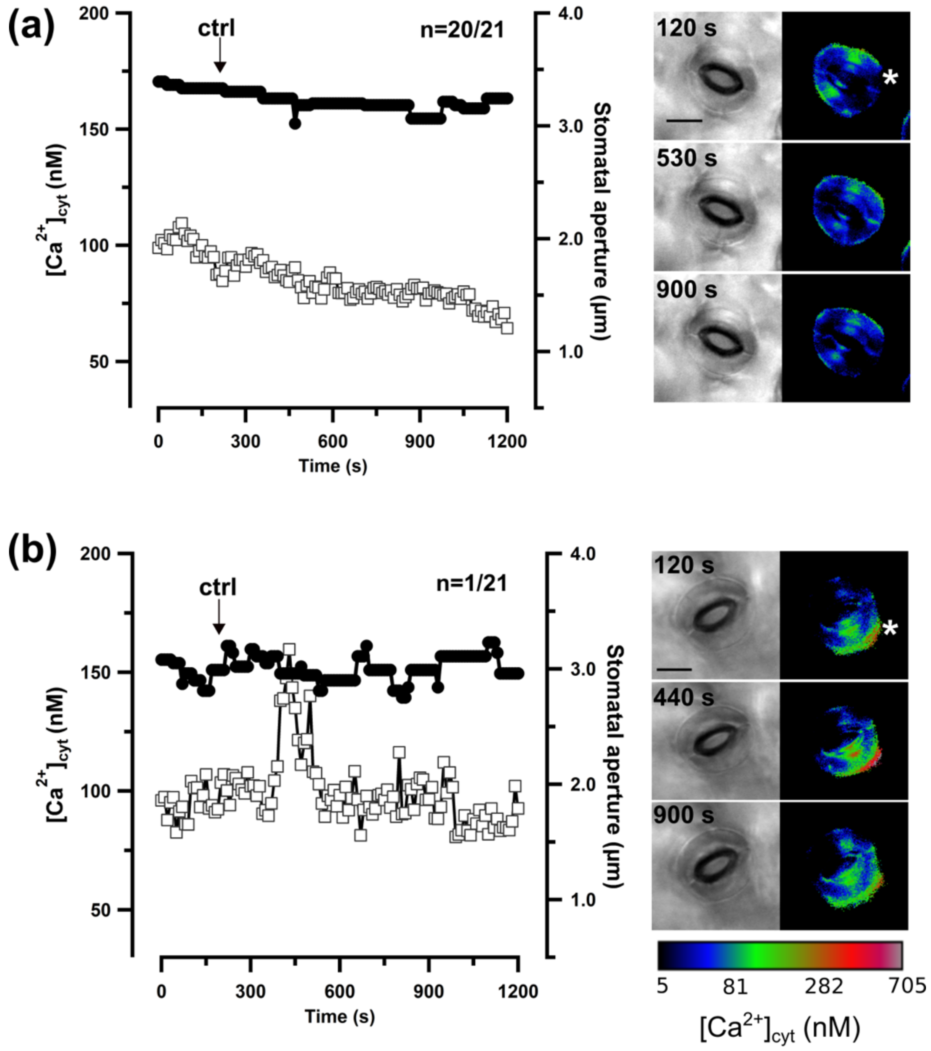

Fig. S5 Current-ejection of benzoic acid only induces Ca^{2+} signals in 1 out of 21 *Arabidopsis thaliana ost1-3* guard cells. (a, b) *left panels*, Time-dependent changes in stomatal aperture (closed circles) and the cytosolic free Ca^{2+} concentration (open squares) in *ost1-3* guard cells, stimulated by current-ejection of benzoic acid (control), as indicated by the arrow. Data are shown for representative guard cells. (a) In 20 out of 21 guard cells, no change occurred in the cytosolic free Ca^{2+} concentration. (b) Current-ejection of benzoic acid was followed by a transient increase of the cytosolic free Ca^{2+} level in 1 out of 21 cells, but nevertheless the stoma remained open. (a, b) *right panels*, Bright field (left image) and pseudo-color (right image) images, showing the stomatal aperture and cytosolic Ca^{2+} concentration in same guard cell, as in the graphs on the left. The images were obtained at three time points, as indicated in the bright field images. The asterisks in the pseudo-color images indicate the position of contact between the current-ejection electrode and the guard cell wall. The calibration bar below the images links the color-code to the cytosolic free Ca^{2+} concentration. Scale bar=10 µm.
