# Supplementary material for: Calcium signals in guard cells enhance the efficiency by which abscisic acid triggers stomatal closure
Source: New Phytol. 2019 Jul 19;224(1):177–87. doi: 10.1111/nph.15985 (PMC6771588; doi:10.1111/nph.15985)
Supplement: Supplementary file 2 — Methods S1 Procedures to estimate the ABA concentration in the guard cell wall that was imposed by current‐ejection and calibration of R‐GECO1‐mTurquoise with FURA2. [file NPH-224-177-s002.pdf]

Supporting Information Methods S1 to:

Ca^{2+} signals in guard cells enhance the efficiency by which ABA triggers stomatal closure

By: Shouguang Huang, Rainer Waadt, Maris Nuhkat, Hannes Kollist, Rainer Hedrich and M. Rob G. Roelfsema

Article acceptance date: 3 June 2019

Estimation of ABA concentration in the guard cell wall, imposed by current-ejection

The efficiency by which ABA was current injected into guard cell walls was estimated with use of the fluorescent dye Lucifer Yellow CH (LY) (Stewart, 1978). LY was excited with light from an LED illumination system (435nm, pE-4000, CoolLED, https://www.coolled.com) filtered by a bandpass filter of 430/24 nm (ET 430/24 Chroma Technologies, www.chroma.com). The emission signal was passed through a dichroic mirror (499 nm LP) and filtered by a 520/30 nm bandpass filter (520/30 nm, Brightline, Semrock, http://www.semrock.com). The signal of charge multiplying CDD camera (QuantEM, Photometrics, http://www.photometrics.com) was calibrated to the LY concentration, by pressure ejection of a range of LY concentrations from a glass capillary (inner diameter, 0.58 mm; outer diameter, 1.0 mm; Hilgenberg, http://www.hilgenberg-gmbh.com), of which the tip was broken back to an opening of 1 µm. A linear relation was found between the concentration of LY and the fluorescence signal detected at the tip of the capillary (Fig. S1a).

In the next step, sharp microelectrodes were used to current-eject LY into the guard cell wall, using the same conditions as for ABA (Fig. 1b). Instead of ABA, the tip of the microelectrodes was filled with 50 µM LY, while the remaining of the electrode was filled with 300 mM KCl. Application of -0.8 nA for 30 s caused a local increase of the LY concentration to 4 µM (SE=0.43, n=12), which decreased exponentially with distance from the site of ejection (Video S2, Fig. S1b and c). These LY data were used to estimate the ABA concentration that was imposed to guard cells by current ejection. The ratio between the flux of ABA and LY, injected into the guard cell wall, is given by Eq. S1;

$$\frac{J_{ABA}}{J_{LY}} = \frac{[ABA]\, Vd_{ABA}}{[LY]\, Vd_{LY}} \qquad (Eq. S1)$$

the concentration of dissociated ABA^- is 21 µM, assuming a pKa of 4.8 (www.chemicalbook.com), while LY will be fully dissociated at a concentration of 50 µM (Stewart, 1978) and Vd_{ABA} and Vd_{LY} represent the drift velocity of ABA and LY. The drift velocity can be calculated with Eq. S2;

$$Vd = \mu_q\, E \qquad (Eq. S2)$$

in which μ_q is the electrical mobility of an ion and E the electrical field that acts on the ion. If an identical electrical field is assumed to act on ABA and LY, the ratio in drift velocity will be given by Eq. S3.

$$\frac{\mu_{q(ABA)}}{\mu_{q(LY)}} = \frac{q_{(ABA)}\ \mu_{(ABA)}}{q_{(LY)}\ \mu_{(LY)}} \qquad (Eq.S3)$$

in which q represents the number of elementary charges (q=1 for ABA and q=2 for LY), and μ the chemical mobility that can be calculated from the diffusion constants of ABA (6 10^{-10} m^2 s^{-1}) (Slovik *et al.*, 1992) and LY (5 10^{-10} m^2 s^{-1}) (Imanaga *et al.*, 1987) using Eq. S4;

$$\mu = \frac{D}{K_b\ T} \qquad (Eq.S4)$$

in which K_b is Bolzmann's constant and T the absolute temperature.

Based on these considerations, a ratio J_{ABA}/J_{LY} = 0.25 was calculated and we used this value to estimate the ABA concentration that was applied in the guard cell wall (Fig. S1d).

Calibration of the genetically encoded calcium reporter R-GECO1-mTurquoise

The emission ratio signal of R-GECO1-mTurquoise (RG-mT) was calibrated, with use of the excitation ratio values of FURA2, which was current injected into guard cells expressing RG-mT (Fig. S2a). FURA2 has been intensively used to study cytosolic Ca^{2+} signals in guard cells (McAinsh *et al.*, 1990; Grabov & Blatt, 1998; Levchenko *et al.*, 2005) and the procedure used to calibrate FURA2 is described below. On average, the RG-mT ratio (calculated from the emission signals at 628 and 475 nm) approximated a value of 1 at control conditions (Fig. S2a and b). The upper guard cell of the stoma in Fig. S2a was impaled with a double-barreled electrode, of which the tip of one barrel was filled with 10 mM FURA2 and the remaining with 300 mM KCl, while the second barrel was filled solely with 300 mM KCl. FURA2 was injected into the guard cell, by application of a -1 nA current through the FURA2 containing barrel, for approximately 100 s. The ratios of RG-mT and FURA2 simultaneously increased during FURA2 injection and slowly returned to basic levels thereafter (Fig. S2a and b). The RG-mT ratio signals were plotted with those of FURA2 (Fig. S2c) against the Ca^{2+} concentration calculated from FURA2 ratio signal (see below). A Hill function was fitted to the relation between the RG-mT ratio and Ca^{2+} concentration. This revealed a Hill coefficient of 0.81 and a Kd of 401 nM (SE=17). The obtained Kd value is very close to the values of 482 and 449 nM that were determined for R-GECO1 in vitro (Zhao *et al.*, 2011; Akerboom *et al.*, 2013). We therefore used the obtained relationship between the RG-mT ratio and cytosolic Ca^{2+} concentration (Fig. S2c), to determine the cytosolic Ca^{2+} level of guard cells.

Calibration of FURA2

FURA2 was repetitively excited with UV-flashlight pulses of 200 ms, at 345, 360 and 390 nm, using a time interval of 5 s (VisiChrome high-speed polychromator system, Visitron Systems, http://www.visitron.de), as described previously (Levchenko *et al.*, 2008; Stange *et al.*, 2010; Voss *et al.*, 2016). The emission signal of FURA2 was passed through a dichroic mirror (FT 410; Zeiss, Germany) and a 510 nm bandpass filter (D510/40M, Chroma Technology Corp) and recorded with the Visiview software package (Visitron). The FURA2 F345/F390 excitation ratio was converted into the cytosolic free calcium according to the equation (Grynkiewicz *et al.*, 1985);

$$[\mathrm{Ca}^{2+}]_{\mathrm{free}} = K_d \frac{(R - R_{min})F_{min}}{(R_{max} - R)F_{max}}$$

where Kd stands for the dissociation constant of FURA2 for Ca^{2+} (270 nM), R represents the FURA2 emission ratio of signals obtained at 345 and 390 nm, R_{min} and R_{max} correspond to minimal and maximal FURA2 ratio, respectively, and F_{min} and F_{max} the corresponding fluorescence intensity, measured at 390 nm.

The value of R_{min} was determined with epidermal strips kept in a bath solution containing 10 mM CsCl, 1 mM $LaCl_3$ and 10 mM MES-BTP, pH 6.0, to block K^+- and non-selective cation channels. Guard cells were impaled with double-barreled electrodes; the tip of one barrel was filled with 10 mM FURA2, while the other contained 250 mM BAPTA and the remaining of both barrels was filled with 300 mM KCl. After loading of FURA2, BAPTA was injected into the cytosol with currents ranging from -150 to -300 pA (Fig. S2c). BAPTA-injection caused a decrease for the F345/F390 ratio to an average value of 0.23 (SE=0.002, n=6).

R_{max} was determined with guard cells that were loaded with FURA2 just as described above, but the second barrel of the electrode was filled with 300 mM KCl, instead of BAPTA. The epidermal strips were kept in a bath solution with 10 mM KCl, 1 mM $CaCl_2$ and 10 mM MES-BTP pH 6.0. Guard cells were clamped for 30s to -300 mV to get a massive increase of the cytosolic free Ca^{2+} concentration and an increase of the F345/F390 ratio to an average value of 5.34 (SE=0.16, n=6).

References

Akerboom J, Carreras Calderon N, Tian L, Wabnig S, Prigge M, Tolo J, Gordus A, Orger MB, Severi KE, Macklin JJ, et al. 2013. Genetically encoded calcium indicators for multi-color neural activity imaging and combination with optogenetics. *Front. mol. neurosci.* **6**: 2.

Grabov A, Blatt MR. 1998. Membrane voltage initiates Ca^{2+} waves and potentiates Ca^{2+} increases with abscisic acid in stomatal guard cells. *Proceedings of the National Academy of Sciences of the United States of America* **95**(8): 4778-4783.

Grynkiewicz G, Poenie M, Tsien RY. 1985. A new generation of Ca^{2+} indicators with greatly improved fluorescence properties. *Journal of Biological Chemistry* **260**(6): 3440-3450.

Imanaga I, Kameyama M, Irisawa H. 1987. Cell-to-cell diffusion of fluorescent dyes in paired ventricular cells. *American Journal of Physiology* **252**(1): H223-H232.

Levchenko V, Guinot DR, Klein M, Roelfsema MRG, Hedrich R, Dietrich P. 2008. Stringent control of cytoplasmic Ca^{2+} in guard cells of intact plants compared to their counterparts in epidermal strips or guard cell protoplasts. *Protoplasma* **233**(1-2): 61-72.

Levchenko V, Konrad KR, Dietrich P, Roelfsema MRG, Hedrich R. 2005. Cytosolic abscisic acid activates guard cell anion channels without preceding Ca^{2+} signals. *Proceedings of the National Academy of Sciences of the United States of America* **102**(11): 4203-4208.

McAinsh MR, Brownlee C, Hetherington AM. 1990. Abscisic acid-induced elevation of guard-cell cytosolic Ca^{2+} precedes stomatal closure. *Nature* **343**(6254): 186-188.

Slovik S, Baier M, Hartung W. 1992. Compartmental distribution and redistribution of abscisic-acid in intact leaves .1. Mathematical formulation. *Planta* **187**(1): 14-25.

Stange A, Hedrich R, Roelfsema MRG. 2010. Ca^{2+}-dependent activation of guard cell anion channels, triggered by hyperpolarization, is promoted by prolonged depolarization. *Plant Journal* **62**(2): 265-276.

Stewart WW. 1978. Functional connections between cells as revealed by dye-coupling with a highly fluorescent naphthalimide tracer. *Cell* **14**(3): 741-759.

Voss LJ, Hedrich R, Roelfsema MRG. 2016. Current injection provokes rapid expansion of the guard cell cytosolic volume and triggers Ca^{2+} signals. *Molecular Plant* **9**(3): 471-480.

Zhao YX, Araki S, Jiahui WH, Teramoto T, Chang YF, Nakano M, Abdelfattah AS, Fujiwara M, Ishihara T, Nagai T, et al. 2011. An expanded palette of genetically encoded Ca^{2+} indicators. *Science* **333**(6051): 1888-1891.
