# Supplementary material for: Calcium signals in guard cells enhance the efficiency by which abscisic acid triggers stomatal closure
Source: New Phytol. 2019 Jul 19;224(1):177–87. doi: 10.1111/nph.15985 (PMC6771588; doi:10.1111/nph.15985)
Supplement: Supplementary file 3 — Videos S1 Stomatal closure induced by current‐ejection of ABA. Videos S2 Current‐ejection of Lucifer Yellow CH (LY) into the wall of an Arabidopsis guard cell. Videos S3 Calibration of R‐GECO1‐mTuquiose (RG‐mT) with FURA2. Videos S4 ABA‐induced rise in the cytosolic Ca2+ concentration of a guard cell, during stomatal closure. Videos S5 ABA‐induced rise in the cytosolic Ca2+ concentration of a guard cell, before stomatal closure. Videos S6 ABA‐induced stomatal closure in the absence of a cytosolic Ca2+ signal in the guard cells. Videos S7 Voltage‐induced Ca2+ signals in an Arabidopsis guard cell. Videos S8 ost1‐3 stoma exposed to ABA, which did neither evoke stomatal closure, nor Ca2+‐signals. Videos S9 ABA‐induced Ca2+‐signals in an ost1‐3 stoma that were not linked to stomatal closure. Videos S10 Voltage‐induced Ca2+ signals in an ost1‐3 guard cell. [file NPH-224-177-s003.zip › nph15985_VideoLegend.pdf]

Supporting Information Legends to Videos S1-S10

Ca^{2+} signals in guard cells enhance the efficiency by which ABA triggers stomatal closure

By: Shouguang Huang, Rainer Waadt, Maris Nuhkat, Hannes Kollist, Rainer Hedrich and M. Rob G. Roelfsema

Article acceptance date: 3 June 2019

Video S01. Stomatal closure induced by current-ejection of ABA. Transmitted light video of an *Arabidopsis* stoma, of which the lower guard cell was subsequently stimulated by current-ejection of benzoic acid (control, 11 min) and ABA (33 min). Note that the lower guard cell becomes flattened after stimulation with ABA, whereas the upper guard cell remains curved. Scale bar = 10 µm, see also Fig. 1b.

Video S02. Current-ejection of Lucifer Yellow CH (LY) into the wall of an *Arabidopsis* guard cell. Transmitted infrared light (left panel) and fluorescence signal (right panel, color code indicated in bar next to the panel). The wall of the guard cell on the right was loaded with LY, by current-ejection through a single-barreled electrode of which the tip was filled with 50 µM LY. After 130 s (indicated in the upper right corner) a current of -0.8 nA was applied for 30 s, which caused a local rise in fluorescence intensity. Scale bar = 10 µm, see also Fig. S1b and c.

Video S03. Calibration of R-GECO1-mTuquiose (RG-mT) with FURA2. Fluorescence ratio signals of RG-mT (upper panel, color code indicated in upper bar) and FURA2 (lower panel, color code indicated in lower bar). After 120 s (time indicated in upper left corner) the upper guard cell was loaded with FURA2, which caused a simultaneous increase of the RG-mT and FURA2 ratio signals, which slowly decreased again after 180 s. Scale bar = 10 µm, see also Fig. S2a and b.

Video S04. ABA-induced rise in the cytosolic Ca^{2+} concentration of a guard cell, during stomatal closure. Transmitted infrared light (upper panel) and color coded cytosolic Ca^{2+} concentration (lower panel, color code is linked to the free Ca^{2+} concentration (nM) in bar next to the panel), of an *Arabidopsis* stoma, of which the guard cell on the right was stimulated by current-ejection of ABA after 200 s (time indicated in the upper right corner). Note that an increase of the cytosolic Ca^{2+} concentration occurred during closure of the stomatal pore. Scale bar = 10 µm, see also Fig. 2a.

Video S05. ABA-induced rise in the cytosolic Ca^{2+} concentration of a guard cell, before stomatal closure. Transmitted infrared light (upper panel) and color coded cytosolic Ca^{2+} concentration (lower panel, color code is linked to the free Ca^{2+} concentration (nM) in bar next to the panel), of an *Arabidopsis* stoma, of which the lower guard cell was stimulated by current-ejection of ABA after 200 s (time indicated in the upper right corner). Note that an increase of the cytosolic Ca^{2+} concentration occurred before the stomatal pore started to close. Scale bar = 10 µm, see also Fig. 2b.

Video S06. ABA-induced stomatal closure in the absence of a cytosolic Ca^{2+} signal in the guard cells. Transmitted infrared light (upper panel) and color coded cytosolic Ca^{2+} concentration (lower panel, color code is linked to the free Ca^{2+} concentration (nM) in bar next to the panel), of an *Arabidopsis* stoma, of which the lower guard cell was stimulated by current-ejection of ABA after 200 s (time indicated in the upper right corner). Note that the stomatal pore closed, even though no increase of the cytosolic Ca^{2+} concentration occurred. Scale bar = 10 µm, see also Fig. 2c.

Video S07. Voltage-induced Ca^{2+} signals in an *Arabidopsis* guard cell. Color coded cytosolic Ca^{2+} concentration (color code is linked to the free Ca^{2+} concentration (nM) in bar next to the panel) of an *Arabidopsis* stoma, of which the guard cell on the right was impaled with a double-barreled microelectrode and stimulated with 10 s voltage pulses from -100, to -180, -200 and -220 mV, as indicated next to the guard cell. Note that the pulses of -200 and -220 mV caused a transient rise in the cytosolic Ca^{2+} concentration. Scale bar = 10 µm, see also Fig. 4b.

Video S08. ABA-insensitive *ost1-3* stoma. Transmitted infrared light (left panel) and color coded cytosolic Ca^{2+} concentration (right panel, color code is linked to the free Ca^{2+} concentration (nM) in bar next to the panel) of an *ost1-3* stoma, of which the guard cell on the right was stimulated by current-ejection of ABA after 200 s (time indicated in the upper right corner). Note that ABA neither induced stomatal closure, nor a change in the cytosolic Ca^{2+} concentration. Scale bar = 10 µm, see also Fig. 6a.

Video S09. ABA-induced Ca^{2+}-signals in an ost1-3 stoma that were not linked to stomatal closure. Transmitted infrared light (left panel) and color coded cytosolic Ca^{2+} concentration (right panel, color code is linked to the free Ca^{2+} concentration (nM) in bar next to the panel), of an *ost1-3* stoma, of which the lower guard cell was stimulated by current-ejection of ABA after 200 s (time indicated in the upper right corner). Note that ABA did not induce stomatal closure, even though transient changes in the cytosolic Ca^{2+} concentration occurred after hormone application. Scale bar = 10 µm, see also Fig. 6b.

Video S10. Voltage-induced Ca^{2+} signals in an *ost1-3* guard cell. Color coded cytosolic Ca^{2+} concentration (color code indicated in bar next to the panel) of an *ost1-3* stoma, of which the guard cell on the right was impaled with a double-barreled microelectrode and stimulated with 10 s voltage pulses from -100, to -180, -200 and -220 mV, as indicated next to the guard cell. Note that all three voltage pulses caused a transient rise in the cytosolic Ca^{2+} concentration. Scale bar = 10 µm, see also Fig. 7a and b.
